# Development of a Toll-Like Receptor-Based Gene Signature That Can Predict Prognosis, Tumor Microenvironment, and Chemotherapy Response for Hepatocellular Carcinoma

**DOI:** 10.3389/fmolb.2021.729789

**Published:** 2021-09-21

**Authors:** Lixia Liu, Bin Liu, Jie Yu, Dongyun Zhang, Jianhong Shi, Ping Liang

**Affiliations:** ^1^ Department of Interventional Ultrasound, Chinese PLA General Hospital, Medical School of Chinese PLA, Beijing, China; ^2^ Department of Ultrasound, Affiliated Hospital of Hebei University, Baoding, China; ^3^ Central Laboratory, Hebei Key Laboratory of Cancer Radiotherapy and Chemotherapy, Affiliated Hospital of Hebei University, Institute of Life Science and Green Development, Hebei University, Baoding, China

**Keywords:** toll-like receptor signaling pathway, hepatocellular carcinoma, prognosis, tumor microenvironment, chemotherapy response, MAP2K2

## Abstract

**Objective:** Emerging evidence highlights the implications of the toll-like receptor (TLR) signaling pathway in the pathogenesis and therapeutic regimens of hepatocellular carcinoma (HCC). Herein, a prognostic TLR-based gene signature was conducted for HCC.

**Methods:** HCC-specific TLRs were screened in the TCGA cohort. A LASSO model was constructed based on prognosis-related HCC-specific TLRs. The predictive efficacy, sensitivity, and independency of this signature was then evaluated and externally verified in the ICGC, GSE14520, and GSE76427 cohorts. The associations between this signature and tumor microenvironment (stromal/immune score, immune checkpoint expression, and immune cell infiltrations) and chemotherapy response were assessed in HCC specimens. The expression of TLRs in this signature was verified in HCC and normal liver tissues by Western blot. Following si-MAP2K2 transfection, colony formation and apoptosis of Huh7 and HepG2 cells were examined.

**Results:** Herein, we identified 60 HCC-specific TLRs. A TLR-based gene signature (MAP2K2, IRAK1, RAC1, TRAF3, MAP3K7, and SPP1) was conducted for HCC prognosis. High-risk patients exhibited undesirable outcomes. ROC curves confirmed the well prediction performance of this signature. Multivariate Cox regression analysis demonstrated that the signature was an independent prognostic indicator. Also, high-risk HCC was characterized by an increased immune score, immune checkpoint expression, and immune cell infiltration. Meanwhile, high-risk patients displayed higher sensitivity to gemcitabine and cisplatin. The dysregulation of TLRs in the signature was confirmed in HCC. MAP2K2 knockdown weakened colony formation and elevated apoptosis of Huh7 and HepG2 cells.

**Conclusion:** Collectively, this TLR-based gene signature might assist clinicians to select personalized therapy programs for HCC patients.

## Introduction

Hepatocellular carcinoma (HCC) represents the most frequent visceral neoplasm, occupying 70–90% of all primary liver cancer (Craig et al., 2020).Currently, surgery, transplantation, and percutaneous ablations have become major therapeutic strategies against HCC (Piñero et al., 2020; Yang et al., 2020). Hepatitis B and C viral infections are the main risk factors of HCC (Sagnelli et al., 2020). This neoplasm is characterized by complex heterogeneity and high recurrence (Yang and Heimbach, 2020). High-risk subjects with potentially undesirable outcomes are required to be monitored, and timely and effective therapeutic strategies should be adopted for prolonging survival duration and improving their quality of life (Liu et al., 2020). Hence, an in-depth understanding in the precise molecular mechanisms of HCC pathogenesis and progress is of importance for enabling prognosis prediction and individualized therapy.

Toll-like receptors (TLRs) are a family of transmembrane signaling receptors, which may activate the innate immune system that is involved in maintaining homeostasis in the liver by recognizing pathogen-associated molecular patterns (Zou et al., 2016). TLRs enable recognizing foreign pathogens like HBV, thereby inducing innate immunity (Campisano et al., 2019). Emerging evidence has suggested that the TLR signaling pathway in the liver could assist the illustration of the mechanism of liver carcinogenesis, as well as to offer feasible novel therapeutic targets against HCC (Sasaki et al., 2020). Hence, this study screened HCC-specific TLRs as well as developed and externally verified a TLR-based gene signature for HCC. This signature enabled predicting survival outcomes and was in relation to tumor microenvironment (TME) and responses to chemotherapy drugs gemcitabine and cisplatin. Our experimental verification confirmed the dysregulation of TLRs in the signature and silencing MAP2K2 weakened colony formation and elevated apoptosis of HCC cells. Thus, our findings might assist clinicians to select personalized therapy programs for HCC patients and to offer insights into the mechanisms of HCC.

## Materials and Methods

### Data Collection

RNA sequencing profiles (FPKM format) and matched clinicopathologic characteristics of 368 HCC samples were retrieved from the Cancer Genome Atlas (TCGA) database (https://portal.gdc.cancer.gov/repository) through Genomic Data Commons (https://portal.gdc.cancer.gov/) using TCGA biolinks package (Colaprico et al., 2016) on May 16, 2020. Meanwhile, the gene expression profiling of 49 normal liver tissue specimens were also collected. RNA-seq profiles and corresponding clinicopathologic features of 231 HCC specimens were downloaded from the International Cancer Genome Consortium portal (ICGC/TCGA Pan-Cancer Analysis of Whole Genomes Consortium, 2020)(https://dcc.icgc.org/projects/LIRI-JP). The GSE14520 (Roessler et al., 2010) and GSE76427 (Grinchuk et al., 2018) datasets were retrieved from the Gene Expression Omnibus (GEO) repository (https://www.ncbi.nlm.nih.gov/gds/). The GSE76427 dataset contained gene expression data and survival information of 242 HCC patients. Furthermore, the GSE76427 dataset included microarray expression profiling and prognostic data of 115 HCC patients. Our research followed the TCGA, ICGC, and GEO data access policies as well as publication guidelines. For TCGA-HCC, FPKM format was converted to TPM value. For the GSE14520 data from the Affymetrix platform, the raw “CEL” file was background-checked and quantile normalized with a robust multi-array averaging method using affy and simple affy packages. For the GSE76427 data from the Illumina platform, the normalized matrix file was directly downloaded. The gene set of the TLR family was collected from the Kyoto Encyclopedia of Genes and Genomes (KEGG) pathway database (https://www.kegg.jp/kegg) (Kanehisa and Goto, 2000) (Supplementary Table 1). The original code of bioinformatics analysis is listed in Supplementary table 2.

### Differential Expression Analysis

For identifying HCC-specific TLRs, differentially expressed genes (DEGs) between 368 HCC and 49 normal liver tissues were screened in the TCGA cohort utilizing limma package (Ritchie et al., 2015). The screening criteria of HCC-specific TLRs were |log2 fold-change|>2 and adjusted *p* < 0.05.

### Developing and Validating a TLR-Based Gene Signature for HCC Prognosis

HCC-specific TLRs were utilized for univariate Cox regression analyses of overall survival (OS) in the TCGA cohort. For minimizing the risk of overfitting, TLRs with *p* < 0.05 were retained for constructing a least absolute shrinkage and selection operator (LASSO) Cox regression model. Utilizing glmnet package, variable selection and shrinkage were carried out (Engebretsen and Bohlin, 2019). The penalty parameter (λ) was identified by 10-fold cross-verification after the minimum criteria. The TLR-based risk score (RS) of each HCC patient was calculated based on the expression and coefficient of each candidate variables. Utilizing survival ROC package, time-dependent receiver operator characteristic (ROC) curves were depicted for evaluating the predictive power of this model. After stratifying the subjects into high- and low-RS groups according to the median RS, prognostic analyses were presented through Kaplan–Meier curves and log-rank tests. Moreover, the predictive performance of the TLR-based gene signature was externally verified in the ICGC, GSE14520, and GSE76427 cohorts.

### Establishing and Verifying a Prognosis-Related Nomogram

Prognostic independence of the TLR-based signature was determined from other clinical features utilizing uni- and multivariate Cox regression analyses. The independent prognosis-related indicators were included for building a nomogram in the TCGA and ICGC cohorts with rms package. Time-dependent ROC curves were conducted for assessing the predictive performance of the nomogram on one-, three-, and five-year OS probabilities. The calibration diagrams were presented for verifying the prediction accuracy of this model compared with the actual survival time.

### Gene Set Enrichment Analysis (GSEA)

For exploring the molecular mechanisms underlying the TLR-based gene signature, the gene sets of Kyoto Encyclopedia of Genes and Genomes (KEGG) pathways in C2 were curated from the Molecular Signatures Database (https://www.gsea-msigdb.org/gsea/msigdb) (Liberzon et al., 2015). The GSEA algorithm was employed to compare the enrichment differences of the gene sets between high- and low-risk groups according to the gene expression profiles of HCC in the TCGA dataset (Subramanian et al., 2005). Pathways with FDR<0.05 were significantly enriched.

### Prediction Sensitivity of the TLR-Based Gene Signature by Subgroup Analyses

The distribution of the TLR-based RS among clinicopathologic characteristics (grades and stages) for HCC subjects in the TCGA cohort was compared via Kruskal–Wallis tests. After stratifying the subjects into different subgroups according to clinicopathologic characteristics, prognosis analyses were carried out between high- and low-RS subjects.

### Estimation of Tumor Microenvironment

By the Estimation of STromal and Immune cells in MAlignant Tumours using Expression data (ESTIMATE) method (Yoshihara et al., 2013), immune and stromal scores were determined for inferring the fractions of immune and stromal cells in HCC tissues from the TCGA dataset. By combining immune and stromal scores, tumor purity was then calculated. The mRNA expression of immune checkpoints was calculated in each HCC specimen. The infiltration levels of 28 immune cells were estimated with the single-sample gene set enrichment analysis (ssGSEA) method (Hänzelmann et al., 2013).

### Responses to Chemotherapy Drugs

The responses to chemotherapy drugs including gemcitabine and cisplatin were estimated with the Genomics of Drug Sensitivity in Cancer (GDSC; https://www.cancerrxgene.org/) database (Yang et al., 2013). The half maximal inhibitory concentration (IC50) was quantified with pRRophetic package (Geeleher et al., 2014).

### Patients and Specimens

This study collected 3 cases of HCC and corresponding adjacent tissues from HCC patients who underwent surgical resection at the Affiliated Hospital of Hebei University from January 1, 2021 to May 1, 2021. These subjects were diagnosed as HCC with postoperative pathology. The adjacent tissue was normal liver tissue that was more than 2 cm from the edge of the tumor. All patients did not receive radiotherapy, chemotherapy, or other adjuvant treatments before surgery. All fresh tissues obtained were stored in liquid nitrogen as soon as possible after being obtained. This study was reviewed and approved by the Ethics Committee of Affiliated Hospital of Hebei University (2021013), and the patients’ informed consent was obtained.

### Western Blot

RIPA lysis buffer (Millipore, United States) was utilized for extracting the total protein from tissue specimens. By using the BCA protein quantification kit, the protein concentration was determined. After protein denaturation treatment, SDS–PAGE gel electrophoresis was used to separate cellular proteins. The electrophoresis was stopped according to the prestained marker band. The sample was then transferred to the PVDF membrane. Then, the membrane was blocked by 3% BSA prepared with TBS-T overnight at 4°C. After blocking, the membrane was incubated with diluted primary antibodies against MAP2K2 (1/1,000; ab265586; Abcam, United States), IRAK1 (1/1,000; ab238; Abcam, United States), RAC1 (1/1,000; ab97732; Abcam, United States), TRAF3 (1/1,000; ab23935; Abcam, United States), MAP3K7 (1/1,000; ab25879; Abcam, United States), SPP1 (1/1,000; ab166709; Abcam, United States), and GAPDH (1/1,000; ab8245; Abcam, United States) overnight at 4°C, followed by being incubated with goat anti-rabbit IgG–HRP antibody (Santa, United States) or goat anti-mouse IgG-HRP antibody (Santa, United States) for 2 h at room temperature. The protein was developed by ECL luminescent agent. ImageJ software was utilized for detecting the gray value.

### Cell Culture and Transfection

Huh7 and HepG2 cells were purchased from ATCC (United States), which were cultured in DMEM (ThermoFisher Scientific, United States) containing 100 ml/L fetal bovine serum (FBS; Gibco, United States), 100 U/mL penicillin, and 100 mg/L streptomycin. Transfection was carried out according to the instructions of Lipofectamine^TM^ 2000 reagent (Invitrogen, United States). Huh7 and HepG2 cells were seeded in a 6-well plate and cultured until the cell confluence was 50–70%; 24 h before transfection, FBS was replaced with DMEM containing 100 ml/L FBS without double antibodies. During transfection, 100 nmol siRNAs against MAP2K2 (si-MAP2K2) and 100 nmol siRNA negative control (si-NC) were transfected into Huh7 and HepG2 cells with Lipofectamine^TM^ 2000 transfection reagent. The siRNAs were synthesized by Genecopoeia company (United States). After continuing the culture for 8 h with DMEM containing 100 ml/L FBS without a double antibody, the culture medium was replaced with medium and continued to culture. The cells were collected for 48 h after transfection.

### Quantitative Real-Time Polymerase Chain Reaction

Trizol reagent (Solarbio, Beijing, China) was utilized for extracting total RNA from Huh7 and HepG2 cells. A spectrophotometer was used to measure RNA concentration. The extracted RNA was detected by agarose gel electrophoresis. Reverse transcription of RNA into cDNA was carried out based on the following conditions: at 37°C for 15 min and at 85°C for 5 s. The PikoReal^TM^ RT-PCR detection system (Thermo Fisher, United States), SYBR Premix Ex Taq Ⅱ reagent, and qRT-PCR were used for detecting the mRNA expression of MAP2K2 as follows: at 95 C predenaturation for 5 min, a total of 40 cycles of denaturation at 95 C for 10 s, annealing at 59 C for 30 s, and annealing at 60 C for 30 s. The primer sequences of MAP2K2 and GAPDH included MAP2K2, 5′-CCA​AGG​TCG​GCG​AAC​TCA​AA-3’ (F), 5′-TCT​CAA​GGT​GGA​TCA​GCT​TCC-3’ (R), GAPDH, 5′-GGC​AAG​TTC​AAC​GGC​ACA​G-3’ (F), and 5′-ACG​CCA​GTA​GAC​TCC​ACG​AC-3’ (R). Image-Pro Plus image analysis system was applied for calculating the OD value, with GAPDH as an internal control. The relative expression of MAP2K2 mRNA was determined with the 2^-ΔΔCt^ method.

### Clone Formation Assay

Huh7 and HepG2 cells were seeded in a 6-well plate (1,000 cells per well). After placing the cells in the incubator for 2 weeks, the supernatant was discarded. The cells were stained with crystal violet for 30 min. After being washed with PBS 3 times, the number of colonies was counted.

### Flow Cytometry

An annexin Ⅴ–fluorescein isothiocyanate (FITC)/propidium iodide (PI) apoptosis detection kit (BestBio, Shanghai, China) was utilized for examining cell apoptosis. Huh7 and HepG2 cells were cultured normally to the logarithmic growth phase. After 0.25% trypsinization of the cells, the cell pellet was collected and resuspended in 100 μL PBS. Using the FACSCalibur flow cytometer (Becton Dickinson, United States), the fluorescence signal intensity was tested.

### Statistical Analysis

R language (version 3.5.2) and GraphPad Prism software (version 8.0.1) were applied for statistical analyses. Student’s t-test or the Wilcoxon test was applied for comparing the differences between two groups. The Kruskal–Wallis test was utilized for comparisons between multiple groups. A *p*-value less than 0.05 indicates statistical significance.

## Results

### Identification of HCC-specific TLRs

**FIGURE 1:**
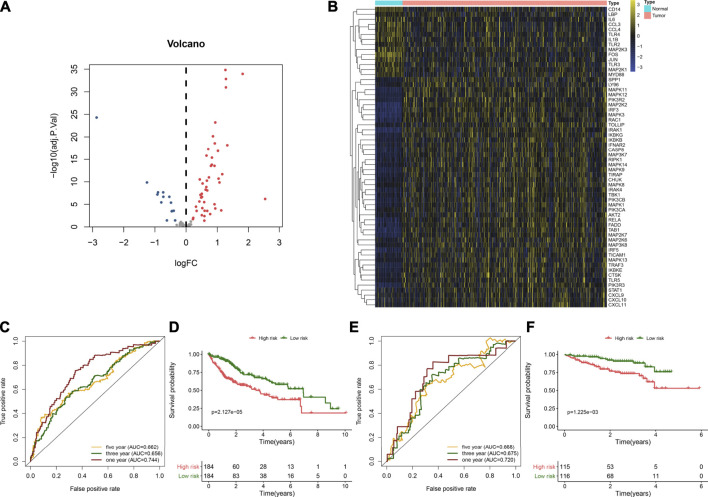
Developing and verifying a TLR-based gene signature in HCC. **(A)** Volcano diagram of the HCC-specific TLRs with |log2fold-change| >2 and adjusted *p* < 0.05 by comparing 368 HCC and 49 normal tissue specimens in TCGA cohort. Blue represents low expression, while red represents high expression. **(B)** Hierarchical clustering analyses of the expression patterns of previous HCC-specific DEGs in 368 HCC and 49 normal samples in TCGA cohort. Yellow indicates high expression, while blue indicates low expression. **(C)** ROC curves of one-, three, and 5-year OS for HCC patients based on the RS in the TCGA cohort. **(D)** Kaplan–Meier curves of high- and low-RS HCC patients in the TCGA cohort. Survival differences were estimated through log-rank tests. **(E)** ROC curves of one-, three-, and five-year OS for HCC subjects according to the RS in the ICGC cohort. **(F)** Kaplan–Meier curves of high- and low-RS HCC subjects in the ICGC cohort.

Here, we collected the expression profiling of 368 HCC and 49 normal liver tissues from the TCGA cohort. With the |log2 fold-change|>2 and adjusted *p* < 0.05, we identified 60 HCC-specific TLRs (Table 1). Among them, 14 TLRs were downregulated, while 46 were upregulated in the HCC tissues in comparison with the normal tissues (Figures 1A,B). This indicated that dysregulation of these TLRs might contribute to HCC progression.

**TABLE 1:** Identification of 60 HCC-specific TLRs in the TCGA cohort.

Gene	Logfold-change	t	*p*-value	Adjusted *p*-value	Genes	Logfold-change	t	*p*-value	Adjusted *p*-value
MAP2K2	1.262,007	14.13751	1.76E-37	1.46E-35	JUN	−0.91985	−5.67973	2.50E-08	6.70E-08
IRAK1	1.813,295	13.85387	2.71E-36	1.12E-34	RIPK1	0.459,955	5.637,478	3.15E-08	8.16E-08
MAPK3	1.277,511	13.54618	5.13E-35	1.42E-33	MAP2K3	−0.53643	−5.45495	8.32E-08	2.04E-07
IRF3	1.275,886	13.06273	4.95E-33	1.03E-31	IL1B	−0.73002	−5.45383	8.37E-08	2.04E-07
FOS	-2.87013	−11.3476	2.95E-26	4.89E-25	MAPK14	0.511,096	5.428,151	9.58E-08	2.27E-07
TAB1	0.936,151	11.03395	4.53E-25	6.27E-24	TBK1	0.485,042	5.362,917	1.35E-07	3.10E-07
RAC1	0.862,191	10.18781	5.84E-22	6.93E-21	SPP1	2.545,628	5.220,422	2.80E-07	6.27E-07
PIK3R3	0.920,451	9.727,353	2.50E-20	2.59E-19	CHUK	0.426,141	4.950,201	1.07E-06	2.34E-06
MAPK11	1.322,901	9.587,574	7.65E-20	7.06E-19	TICAM1	0.580,411	4.878,422	1.51E-06	3.22E-06
MAP2K7	0.709,382	9.322,688	6.20E-19	5.15E-18	CCL4	−0.79546	−4.84886	1.74E-06	3.62E-06
IRF5	1.022618	9.195,042	1.68E-18	1.27E-17	MAP2K1	−0.48377	−4.81614	2.04E-06	4.12E-06
RELA	0.654,615	8.85703	2.24E-17	1.55E-16	CTSK	0.911,693	4.633,718	4.78E-06	9.45E-06
MAPK9	0.82763	8.71183	6.68E-17	4.27E-16	TOLLIP	0.317,776	4.354,871	1.67E-05	3.22E-05
TRAF3	0.826,759	8.204,777	2.76E-15	1.64E-14	TLR5	0.52031	4.18539	3.46E-05	6.53E-05
FADD	0.798,302	8.109,337	5.47E-15	2.87E-14	MAPK13	0.935,497	4.124,942	4.46E-05	8.23E-05
CASP8	0.912,569	8.107,729	5.53E-15	2.87E-14	CXCL10	1.127,512	3.887,076	0.000118	0.000212
MAPK12	1.160,537	7.484,739	4.15E-13	2.03E-12	MYD88	−0.39647	−3.83452	0.000145	0.000256
MAP3K7	0.733,166	7.223,867	2.35E-12	1.08E-11	MAP2K6	0.594,048	3.818,365	0.000154	0.000267
IKBKG	1.04067	7.118,719	4.66E-12	2.03E-11	STAT1	0.67728	3.808,574	0.00016	0.000272
PIK3R2	0.437,359	7.053457	7.10E-12	2.95E-11	AKT2	0.338,982	3.793,519	0.00017	0.000282
PIK3CB	0.666,755	6.941,674	1.45E-11	5.74E-11	TLR3	−0.44792	−3.76962	0.000187	0.000304
CD14	−1.25511	−6.79309	3.71E-11	1.39E-10	IL6	−0.46209	−3.73557	0.000213	0.00034
IKBKE	1.07056	6.786,774	3.86E-11	1.39E-10	LY96	0.773,047	3.350,776	0.000878	0.001374
IRAK4	0.528,799	6.695,029	6.82E-11	2.36E-10	MAP3K8	0.502,467	3.175,544	0.001604	0.002466
TIRAP	0.650,435	6.391,471	4.32E-10	1.43E-09	CXCL11	0.587,711	3.026185	0.002627	0.003964
IKBKB	0.626,264	6.14836	1.80E-09	5.75E-09	MAPK8	0.230,296	2.720,881	0.006777	0.010045
MAPK1	0.680,265	6.074488	2.76E-09	8.48E-09	PIK3CA	0.219,792	2.492,356	0.013069	0.01903
TLR4	−0.73416	−5.90592	7.18E-09	2.13E-08	TLR2	−0.35231	−2.25282	0.024779	0.035459

### Establishing a Robust TLR-Based Signature for HCC Prognosis

Univariate Cox regression analyses were carried out for investigating the associations between HCC-specific TLRs and survival outcomes of HCC in the TCGA cohort. In Table 2, twenty TLRs were distinctly in relation to HCC prognosis. By using the LASSO method, six candidate TLRs were included for establishing a prognostic signature (Supplementary figure 1A, B). The TLR-based RS was determined in each HCC subject as follows: RS = MAP2K2 expression * 0.0335734255703416 + IRAK1 expression * 0.0992603217488045 + RAC1 expression * 0.186397323163475 + TRAF3 expression * 0.112022105244872 + MAP3K7 expression * 0.0570747003763387 + SPP1 expression * 0.0601842657782517. ROC curves were conducted for estimating whether RS could be predictive of HCC prognosis. The AUCs of one-, three- and five-year OS were 0.744, 0.656, and 0.662, indicating that the RS was a robust prognostic signature (Figure 1C). With the median RS, this study separated the HCC patients in the TCGA cohort into two groups. Prognostic analysis showed that high-RS patients displayed depressing OS outcomes in comparison to low-RS patients (*p* = 2.127e-05; Figure 1D).

**TABLE 2:** Univariate Cox regression analyses for prognosis-related HCC-specific TLRs in the TCGA cohort.

TLRs	HR	HR.95L	HR.95H	*p*-value
MAP2K2	1.542,385	1.184,561	2.008297	0.001293
IRAK1	1.465,424	1.206,621	1.779,735	0.000116
MAPK3	1.594,620	1.218,688	2.086517	0.000670
IRF3	1.404,824	1.083381	1.821,639	0.010345
RAC1	1.809,247	1.394,231	2.347,799	8.20E-06
IRF5	1.423,168	1.111,840	1.821,672	0.005084
RELA	1.598,045	1.108,498	2.303,791	0.012008
TRAF3	1.620,687	1.265,701	2.075233	0.000129
FADD	1.396,471	1.085862	1.795,930	0.009276
CASP8	1.374,547	1.084153	1.742,723	0.008608
MAPK12	1.216,302	1.047768	1.411,945	0.010078
MAP3K7	1.473,969	1.132,725	1.918,015	0.003883
PIK3CB	1.454,172	1.124,791	1.880,008	0.004272
IKBKE	1.302,939	1.115,145	1.522,358	0.000861
MAPK1	1.296,421	1.028318	1.634,422	0.028078
IFNAR2	1.419,409	1.068899	1.884,856	0.015502
TBK1	1.353,146	1.011841	1.809,577	0.041416
SPP1	1.131,800	1.074585	1.192,060	2.90E-06
LY96	1.129,772	1.014696	1.257,898	0.026005
TLR2	1.198,885	1.029720	1.395,840	0.019421

### External Validation of the TLR-Based Signature

**FIGURE 2:**
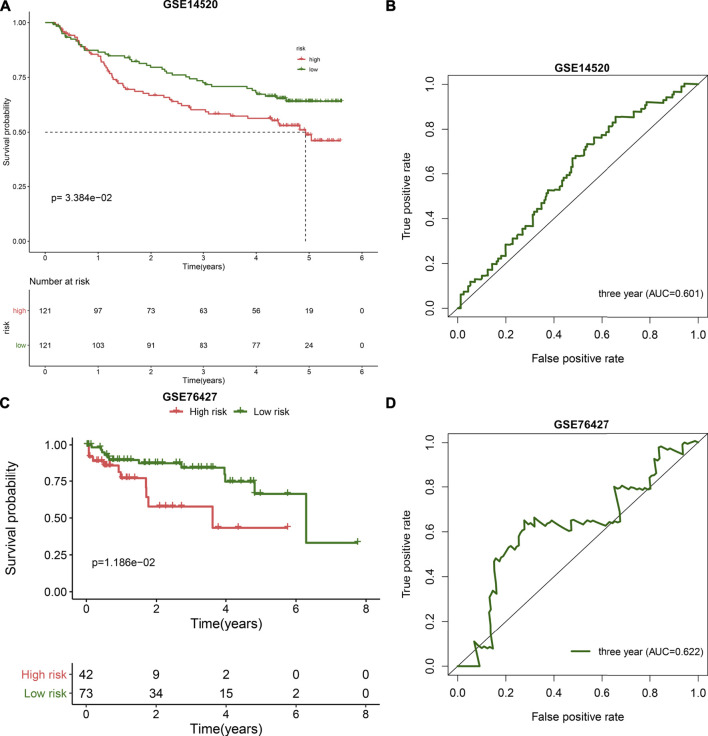
External validation of the TLR-based gene signature in the GSE14520 and GSE76427 datasets. **(A)** Kaplan-Meier curves of high and low RS HCC patients in the GSE14520 dataset. Survival differences were estimated through log-rank tests. **(B)** ROC curve of 3-year OS for HCC patients according to the RS in the GSE14520 dataset. **(C)** Kaplan-Meier curves of high and low RS HCC patients in the GSE76427 dataset. Survival differences were estimated through log-rank tests. **(D)** ROC curve of 3-year OS for HCC patients according to the RS in the GSE76427 dataset.

The predictive performance of the TLR-based signature was externally verified in the ICGC, GSE14520, and GSE76427 cohorts. The AUCs of one-, three-, and five-year OS were 0.720, 0.675, and 0.668 in the ICGC cohort (Figure 1E). In Figure 1F, high RS was indicative of gloomy OS for HCC subjects (*p* = 1.225e-03) in the ICGC cohort. Similarly, in the GSE14520 dataset, patients with high-risk exhibited poorer OS than those with low risk (*p* = 3.384e-02; Figure 2A). And the AUC at three-year OS was 0.601 in the GSE14520 dataset (Figure 2B). Also, we observed the distinct survival advantage for low-risk patients in the GSE76427 dataset (*p* = 1.186e-02; Figure 2C). As shown in Figure 2D, the AUC at three-year OS was 0.622 in the GSE76427 dataset. Following the previous validation, the TLR-based signature was a reliable prognostic predictor of breast cancer.

### Developing a Nomogram by Incorporating Stage and the TLR-Based Signature for Risk Stratification and Survival Prediction of HCC

**FIGURE 3:**
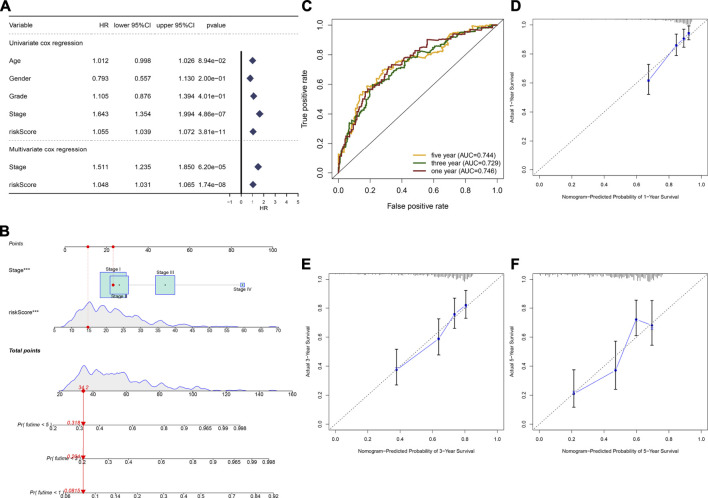
Establishment of a nomogram by incorporating stage and TLR-based RS for HCC prognosis in the TCGA cohort. **(A)** Uni- and multivariate Cox regression analyses of the associations between survival outcomes and age, gender, grade, stage, and TLR RS of HCC patients. **(B)** Constructing a prognostic nomogram that included stage and TLR RS for prediction of one-, three-, and 5-year OS probabilities. **(C)** ROC curves under one-, three-, and 5-year OS for HCC subjects based on the nomogram model. **(D**–**F)** Calibration curves of this model-estimated and observed one-, three-, and 5-year OS probabilities.

In the TCGA cohort, we found that stage (HR: 1.643 (1.354–1.994); *p* = 4.86e-07) and the TLR-based signature (HR: 1.055 (1.039–1.072); *p* = 3.81e-11) displayed significant associations with HCC prognosis according to univariate Cox regression analyses (Figure 3A). Following multivariate Cox regression analyses, stage (HR: 1.511 (1.235–1.850); *p* = 6.20e-05) and the TLR-bases signature (HR: 1.048 (1.031–1.065); *p* = 1.74e-08) were independent prognostic markers of breast cancer. For facilitating clinical application, we established a nomogram by incorporating stage and the TLR-based signature for estimating one-, three-, and five-year OS (Figure 3B). ROC curves confirmed the well-predictive performance for one- (AUC = 0.746), three- (AUC = 0.729), and five-year (AUC = 0.744) OS of HCC patients (Figure 3C). As shown in calibration curves, the model-estimated and observed one-, three-, and five-year OS probabilities were highly close (Figures 3D–F). The well-predictive efficacy of the nomogram was confirmed in the ICGC cohort (Supplementary figure 2A-F).

### Signaling Pathways Involving the TLR Signature

**FIGURE 4:**
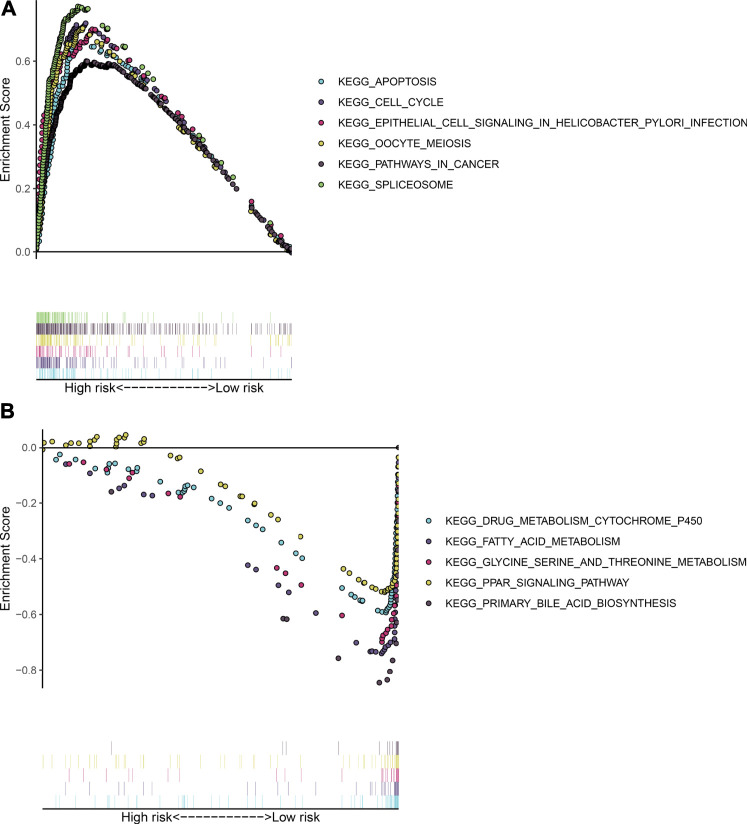
Signaling pathways involving the TLR signature through GSEA. **(A)** KEGG pathways that were activated in HCC subjects with high TLR-based RS: apoptosis, cell cycle, epithelial cell signaling in *Helicobacter pylori* infection, oocyte meiosis, pathway in cancer, and spliceosome. **(B)** KEGG pathways that were activated in HCC subjects with low TLR-based RS: metabolism cytochrome P450, fatty acid metabolism, glycine serine and threonine metabolism, PPAR signaling pathway, and primary bile acid biosynthesis.

Our GSEA results demonstrated that apoptosis (NES = 2.09 and FDR = 0.001), cell cycle (NES = 1.97 and FDR = 0.003), epithelial cell signaling in *helicobacter pylori* infection (NES = 2.08 and FDR = 0.004), oocyte meiosis (NES = 2.16 and FDR < 0.0001), pathway in cancer (NES = 1.99 and FDR = 0.003), and spliceosomes (NES = 1.99 and FDR = 0.003) were distinctly activated in HCC subjects with high TLR-based RS (Figure 4A). Moreover, metabolism of cytochrome P450 (NES = −1.80 and FDR = 0.032), fatty acid metabolism (NES = −1.81 and FDR = 0.036), glycine serine and threonine metabolism (NES = −1.87 and FDR = 0.030), PPAR signaling pathway (NES = −1.65 and FDR = 0.021), and primary bile acid biosynthesis (NES = −1.92 and FDR = 0.038) were markedly activated in HCC subjects with low TLR-based RS (Figure 4B).

### Estimation of the Predictive Sensitivity of the TLR-Based Signature for HCC Prognosis

**FIGURE 5:**
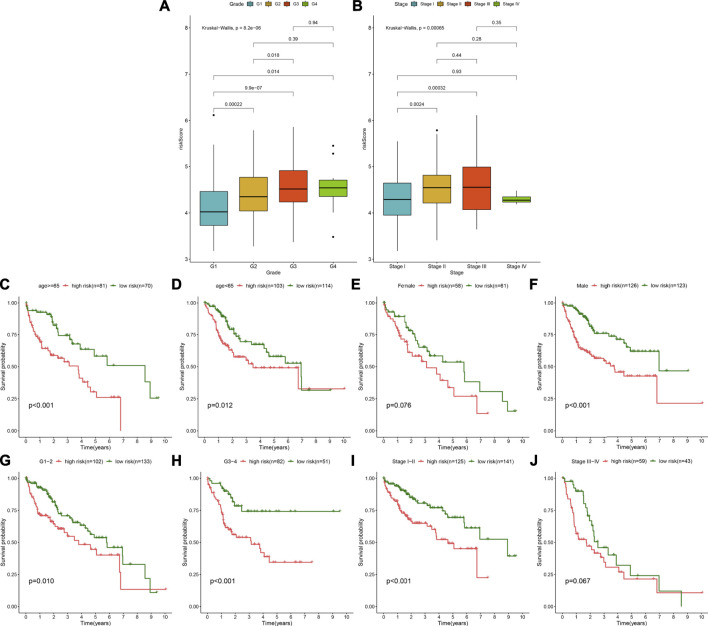
Assessment of the predictive sensitivity of the TLR-based signature for HCC prognosis in the TCGA cohort. **(A)** The distribution of the TLR-based RS in different grades (G1-4) of HCC. **(B)** The distribution of the TLR-based RS in different stages (stage I-IV) of HCC. *p* values were estimated with Kruskal–Wallis tests. **(C**–**J)** Subgroup analysis of prognostic value of the TLR-based signature for HCC patients by Kaplan–Meier curves according to clinicopathologic characteristics: **(C)** age > = 65 and **(D)** age < 65; **(E)** female and **(F)** male; **(G)** grade 1–2 and **(H)** grade 3–4; **(I)** stage I-II and **(J)** stage III-IV. Survival differences were estimated through log-rank tests.

The distribution of the TLR-based RS was analyzed among grades and stages in HCC patients from the TCGA cohort. In Figure 5A, compared with G1, the RS gradually elevated as the grade increased. Furthermore, there were higher RSs in stages II and III than in stage I (Figure 5B). These data indicated that the TLR-based RS was in relation to HCC progression. For analyzing the predictive sensitivity of this signature, we carried out subgroup analyses in the TCGA–HCC cohort. Prognostic analyses showed that high RS was indicative of poorer OS than low RS in each subgroup: ≥ 65 (*p* < 0.001; Figure 5C) and age<65 (*p* = 0.012; Figure 5D); female (*p* = 0.076; Figure 5E) and male (*p* < 0.001; Figure 5F); G1-2 (*p* = 0.010; Figure 5G) and G3-4 (*p* < 0.001; Figure 5H); stage I-II (*p* < 0.001; Figure 5I), and stage III-IV (*p* = 0.067; Figure 5J).

### Associations Between the TLR-Based Signature and TME of HCC

**FIGURE 6:**
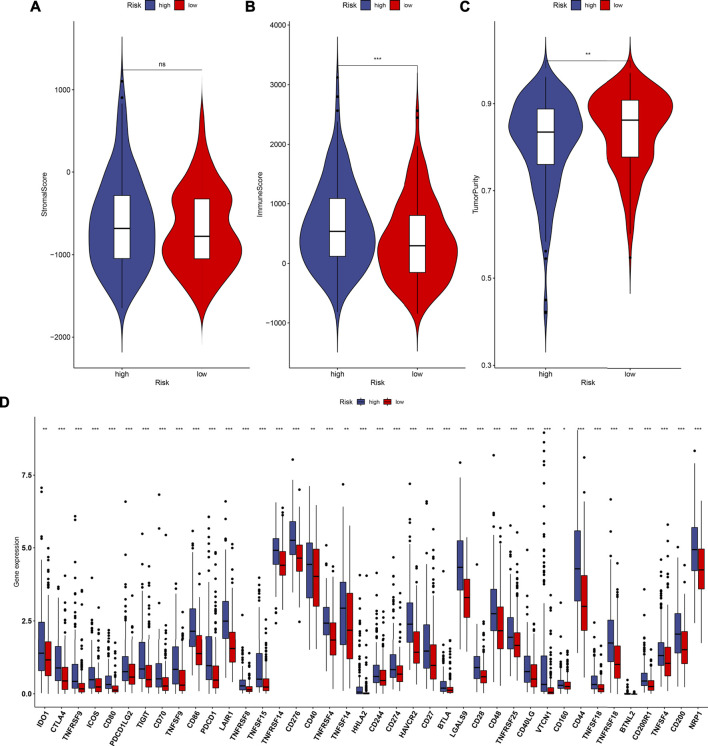
Estimation of the associations between the TLR-based signature and TME of HCC in the TCGA cohort. **(A**–**C)** Comparisons of stromal score, immune score, and tumor purity between high and low TLR-based RS groups using the ESTIMATE method. **(D)** Comparisons of immune checkpoints between high and low TLR-based RS groups. *p* values were estimated with Wilcoxon rank-sum tests. *p < 0.05; **p < 0.01; ***p < 0.001; ns: not significant.

**FIGURE 7:**
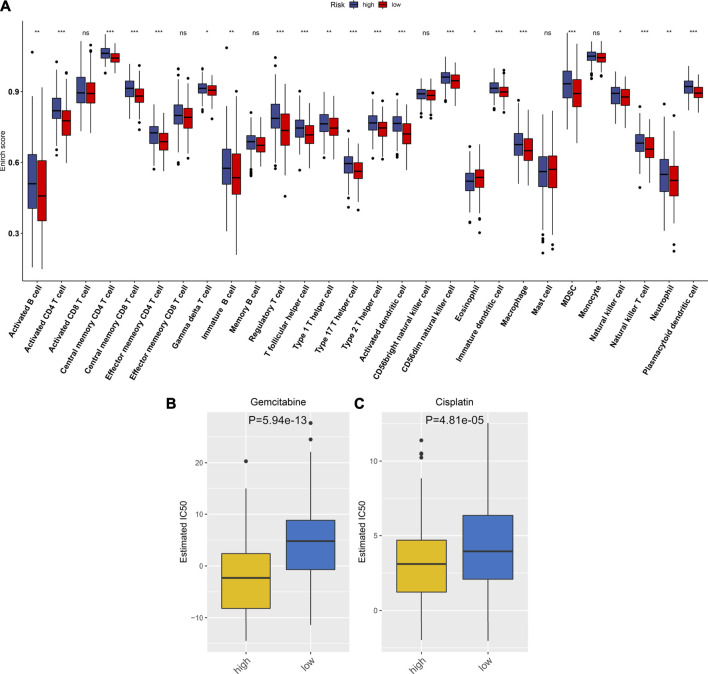
Associations between the TLR-based signature and immune cell infiltrations and chemosensitivity of HCC in the TCGA cohort. **(A)** Comparisons of infiltration levels of immune cells between high and low TLR-based RS groups using the ssGSEA method. **(B, C)** Comparisons of the responses to gemcitabine and cisplatin between high and low TLR-based RS groups by GDSC database. *p* values were estimated utilizing Wilcoxon rank-sum tests. *p < 0.05; **p < 0.01; ***p < 0.001; ns: not significant.

By using the ESTIMATE method, we inferred the infractions of immune and stromal cells in HCC specimens. We found that there was no significant difference in the stromal score between high- and low-RS groups (Figure 6A). In Figure 6B, high-RS samples displayed increased the immune score in comparison to low-RS samples (*p* < 0.001). Also, high RS was characterized by reduced tumor purity compared with low RS (*p* < 0.01; Figure 6C). The differences in immune checkpoint expression were compared between groups. In Figure 6D, we found that high-RS samples displayed an increased mRNA expression of immune checkpoints including CD86, CTLA4, TNFSF15, TNFSF14, TNFRSF18, IDO1, CD27, CD160, CD274, BTLA, TNFRSF9, LAIR1, HHLA2, CD244, CD70, TIGIT, BTNL2, TNFSF9, TNFSF18, NRP1, CD200, CD276, HAVCR2, TNFRSF8, LGALS9, CD28, CD80, PDCD1, CD44, PDCD1LG2, TNFRSF25, TNFRSF14, IDO2, TNFRSF4, CD48, CD40, VTCN1, CD40LG, TNFSF4, and CD200R1. Moreover, the infiltration levels of immune cells were estimated by using the ssGSEA method. As shown in Figure 7A, high-RS specimens were characterized by increased infiltrations of activated B cell, activated CD4 T cell, central memory CD4 T cell, central memory CD8 T cell, effector memory CD4 T cell, gamma delta T cell, immature B cell, regulatory T cell, T follicular helper cell, type 1 T helper cell, type 17 T helper cell, type 2 T helper cell, activated dendritic cell, CD56dim natural killer cell, eosinophil, immature dendritic cell, macrophage, MDSC, natural killer cell, natural killer T cell, neutrophil, and plasmacytoid dendritic cell. The significant associations between the signature and immune cell infiltrations were also found in HCC (Supplementary figure 3A-N).

### Associations Between the TLR-Based Signature and Responses to Chemotherapy Drugs

Using the GDSC database, the responses to chemotherapy drugs were estimated in HCC patients from the TCGA cohort. Here, we analyzed the associations between the TLR-based signature and responses to chemotherapy drugs. Our data showed that high-RS patients displayed lower IC50 values of gemcitabine (*p* = 5.94e-13; Figure 7B) and cisplatin (*p* = 4.81e-05; Figure 7C) than low-RS patients. Hence, high RS predicted better responses to gemcitabine and cisplatin for HCC patients.

### Verification of TLRs in This Prognostic Signature for HCC

**FIGURE 8:**
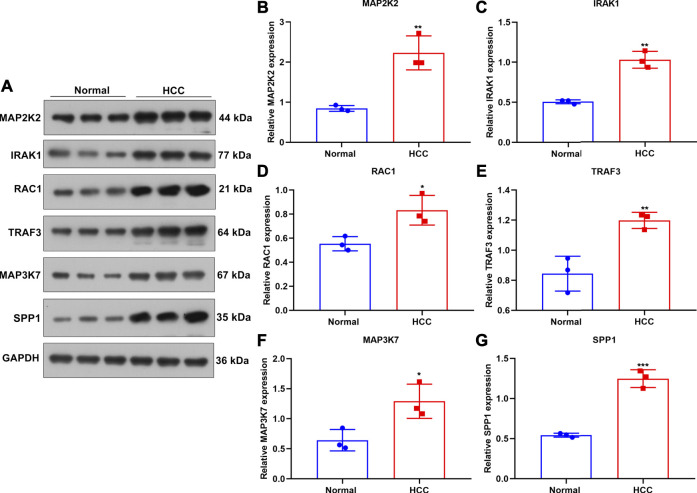
Verification of the expression of genes in the TLR-based signature in HCC and normal tissue specimens. **(A)** Western blot for detecting MAP2K2, IRAK1, RAC1, TRAF, and MAP3K7 proteins in three paired HCC and normal tissues. (B–G) Quantification of the expression of **(B)** MAP2K2, **(C)** IRAK1, **(D)** RAC1, **(E)** TRAF, **(F)** MAP3K7, and **(G)** SPP1 proteins in three paired HCC and normal tissues. Comparisons between groups were evaluated with Student’s t tests. *p < 0.05; **p < 0.01; ***p < 0.001.

This study collected three paired HCC and normal liver tissues. Western blot was applied for examining the expression of TLRs in this prognostic signature in the aforementioned specimens (Figure 8A). Our results confirmed that MAP2K2 (*p* < 0.01; Figure 8B), IRAK1 (*p* < 0.01; Figure 8C), RAC1 (*p* < 0.05; Figure 8D), TRAF3 (*p* < 0.01; Figure 8E), MAP3K7 (*p* < 0.05; Figure 8F), and SPP1 (*p* < 0.001; Figure 8G) were all markedly upregulated in HCC compared with normal liver tissues at the protein levels.

### MAP2K2 Deficiency Weakens Colony Formation Capacity and Enhances Apoptosis in HCC Cells

**FIGURE 9:**
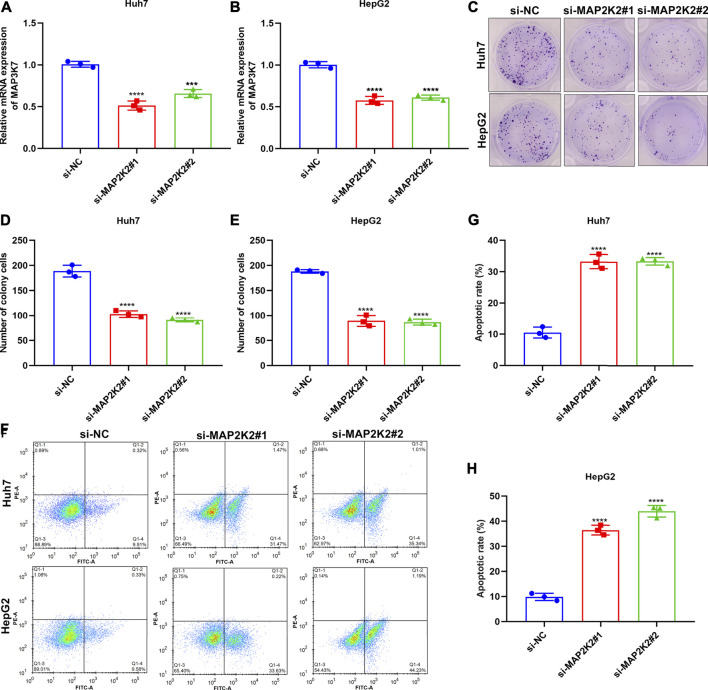
Silencing MAP2K2 weakened colony formation capacity and induced apoptosis of HCC cells. **(A, B)** RT-qPCR of the mRNA expression of MAP2K2 in Huh7 and HeG2 cells with si-MAP2K2 transfection. **(C**–**E)** Colony formation assays of the number of colonies of Huh7 and HeG2 cells with si-MAP2K2 transfection. **(F**–**H)** Flow cytometry assays of the apoptotic levels of Huh7 and HeG2 cells under transfection with si-MAP2K2. *p* values were estimated with ANOVA tests. ***p < 0.001; ****p < 0.0001.

For observing the influence of MAP2K2 on HCC pathogenesis, MAP2K2 expression was markedly decreased in Huh7 and HepG2 cells by si-MAP2K2 transfection (Figures 9A,B). Colony formation capacity was then observed. We found that MAP2K2 deficiency distinctly reduced colony formation of Huh7 and HepG2 cells (Figures 9C–E). Also, MAP2K2 knockdown elevated the apoptotic levels of HCC cells (Figures 9F–H). Thus, MAP2K2 deficiency weakened colony formation capacity and enhanced apoptosis in HCC cells.

## Discussion

As microarrays and high-throughput sequencing step forward (Song et al., 2020; Su et al., 2020; Song et al., 2021; Song and Su, 2021), gene signatures on the basis of mRNA expression profiles exhibit much potential for prediction of HCC outcomes. Several single genes may independently estimate survival outcomes of HCC subjects. For instance, PRIM1 can be used for predicting HCC prognosis, and its upregulation accelerates HCC progression through activation of the AKT/mTOR pathway and UBE2C-induced P53 ubiquitination (Zhu et al., 2021). Moreover, a few prognosis-related signatures according to multiple mRNAs have been conducted for HCC, which might be utilized for preclinical and clinical therapies (Long et al., 2019; Hong et al., 2020; Liang et al., 2020), such as a risk signature based on N^6^-methyladenosine RNA methylation regulators (Bian et al., 2020). Nevertheless, additional signature models should be conducted for accurately predicting HCC prognosis due to complexity and heterogeneity of the neoplasm. Here, we conducted a TLR-based gene signature (including MAP2K2, IRAK1, RAC1, TRAF3, MAP3K7, and SPP1) for HCC prognosis. Following verification, this signature acted as a robust prognostic indicator of HCC.

Cancer is driven by genetic alterations (2020). According to the previous research, the TLRs in this signature exerted key functions in HCC progression. Our Western blot confirmed the dysregulation of the TLRs in HCC. USP21-mediated de-ubiquitination and stabilization of MAP2K2 promotes tumor growth of HCC (Li et al., 2018). MAP2K2 upregulation that is mediated by c-Myb enhances proliferation and invasion of HCC (Zhuang et al., 2019). MAP2K2 knockdown prevents ERK1/2 activation and abolishes HCC progression (Gailhouste et al., 2010). Our data confirmed that MAP2K2 knockdown weakened colony formation capacity and enhanced apoptosis in HCC cells. IRAK1 enhances cancer stemness and weakens the sensitivity to doxorubicin and sorafenib using the AP-1/AKR1B10 axis in HCC (Cheng et al., 2018). Suppression of IRAK1 protects against chronic inflammation–associated HCC (Li et al., 2015). Analyses of immunohistochemistry and RNA-seq profiling reveal that IRAK1 acts as an oncogene and a diagnostic and treatment target against HCC (Ye et al., 2017). IRAK1 heightens cellular proliferative capacity and protects against apoptosis for HCC (Li et al., 2016). RAC1 enhances cancer stemness and self-renewal capacity in HCC (Ran et al., 2019). RAC1 promotes diethylnitrosamine-mediated formation of HCC (Bopp et al., 2015). TRAF3 that is activated by SMAC weakens HCC growth (Ding et al., 2020). MAP3K7 knockdown drives liver fibrosis and HCC via RIPK1 kinase-dependent inflammatory response (Tan et al., 2020). SPP1 possesses the potential for risk stratification and OS prediction of HCC patients (Ouyang et al., 2020).

HCC represents a heterogeneous malignancy, which happens via distinct pathway activation and molecular alterations (Li et al., 2019). Our GSEA demonstrated that high TLR-based RS was distinct in relation to apoptosis, cell cycle, epithelial cell signaling in *Helicobacter pylori* infection, oocyte meiosis, pathway in cancer, and spliceosome, while low TLR-based RS was related to metabolism cytochrome P450; fatty acid metabolism; glycine, serine, and threonine metabolism; PPAR signaling pathway; and primary bile acid biosynthesis. Although epithelial cell signaling in *Helicobacter pylori* infection and oocyte meiosis are not associated with HCC progression, evidence suggests that TLR is involved in modulating the previous pathways (Smith et al., 2011; Gilbert, 2019). The tumor microenvironment including fibroblasts, myofibroblasts, endothelial cells, immune cells, and extracellular matrix plays a critical role in the initiation, growth, and dissemination of HCC (Zhang et al., 2020). Here, we observed that high RS was characterized by a high immune score, immune checkpoint expression, and immune cell infiltration in HCC. TLR, a pattern recognition receptor, is mainly expressed in immune cells containing dendritic cell, macrophage, natural killer cell, and other antigen-presenting cells (Karapetyan et al., 2020). TLR activation induces inflammatory response, thereby resulting in the enhanced uptake and killing of cancer cells and the generation of adaptive immune response (Karapetyan et al., 2020). This also confirmed the implications of the TLR-based signature in immune activation. Despite the progress of therapeutic strategies, patients with intermediate–advanced HCC exhibit low efficacy, partly due to chemoresistance (Zhang et al., 2021). Here, our results showed that high RS was predictive of better responses to gemcitabine and cisplatin for HCC patients.

A nomogram model represents a robust tool for providing probabilistic prediction of persons. Herein, a TLR-based nomogram was conducted, which might estimate HCC outcomes. Our ROC and calibration curves confirmed the favorable predictive performance of this nomogram. Although this study identified a prognostic TLR-based signature that exhibited underlying substantial clinical implications, there are still a few shortcomings. To consider the much heterogeneity of HCC, several candidate TLRs that could affect HCC prognosis might be removed before establishing this prognosis-related TLR-based signature, which might decrease the predictive efficacy of this signature. Furthermore, the mechanism of post-curative relapse and metastases that are the main clinical features that could assist in the interpretation of the relatively poor diagnosis efficacy, except for the follow-up containing post-curative relapse and metastasis information, was lacking in these collected specimens. Additionally, this prognosis-related TLR signature will be observed by an in-depth experiment validation and the clinical applications assessed utilizing a multicenter randomized controlled study.

## Conclusion

Collectively, combining conventional clinicopathologic characteristics, the TLR-based signature displayed the advantage in predicting survival outcomes, TME, and responses to chemotherapeutics in HCC. Hence, this signature had the well prospect in clinical practice.

## Data Availability

The original contributions presented in the study are included in the article/Supplementary Material; further inquiries can be directed to the corresponding author.

## Abbreviations

HCC, hepatocellular carcinoma; TLRs, toll-like receptors; TME, tumor microenvironment; TCGA, The Cancer Genome Atlas; ICGC, International Cancer Genome Consortium; KEGG, Kyoto Encyclopedia of Genes and Genomes; DEGs, differentially expressed genes; OS, overall survival; LASSO, Least Absolute Shrinkage and Selection Operator; RS, risk score; ROC, receiver operator characteristic; GSEA, gene set enrichment analysis; ESTIMATE, Estimation of STromal and Immune Cells in MAlignant Tumours using Expression data; ssGSEA, single-sample Gtene Set Enrichment Analysis; GDSC, Genomics of Drug Sensitivity in Cancer; IC50, half maximal inhibitory concentration; qRT-PCR, quantitative real-time polymerase chain reaction.
