# Supplementary figures and images for: Development of a Toll-Like Receptor-Based Gene Signature That Can Predict Prognosis, Tumor Microenvironment, and Chemotherapy Response for Hepatocellular Carcinoma
Source: Front Mol Biosci. 2021 Sep 21;8:729789. doi: 10.3389/fmolb.2021.729789 (PMC8490642; doi:10.3389/fmolb.2021.729789)

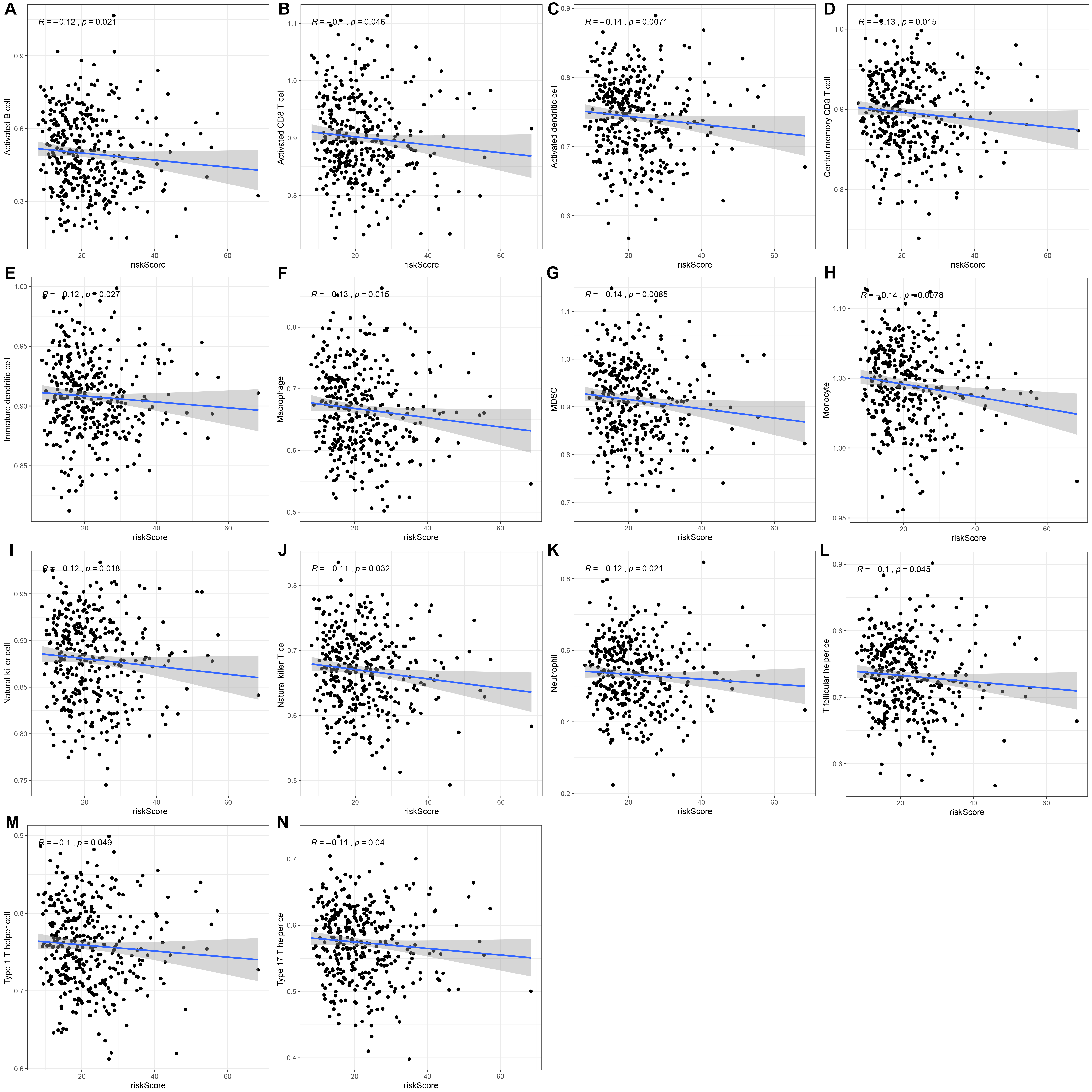

Supplement: Supplementary file 1 [file Image3.TIF]

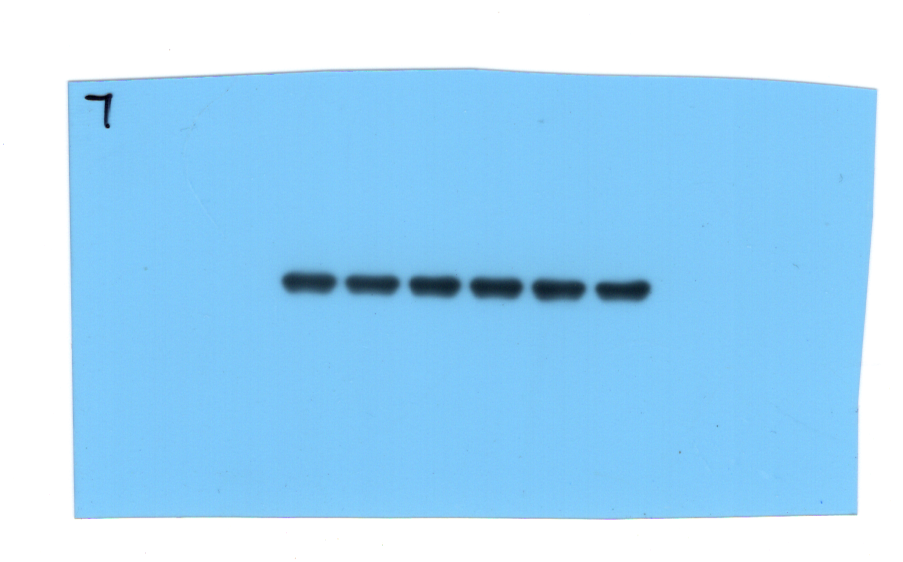

Supplement: Supplementary file 2 [file DataSheet1.ZIP › Original Source Data/Figure 8/Figure 8A-Western blot/GAPDH-36kDa.tif]

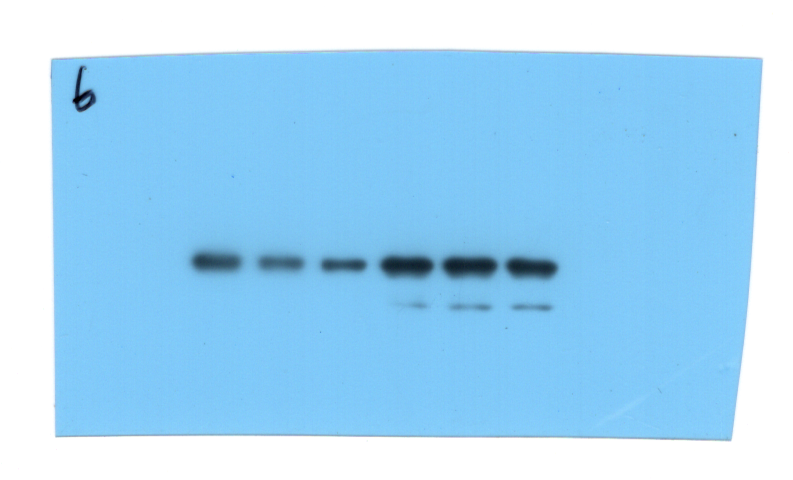

Supplement: Supplementary file 2 [file DataSheet1.ZIP › Original Source Data/Figure 8/Figure 8A-Western blot/IRAK1-77kDa.tif]

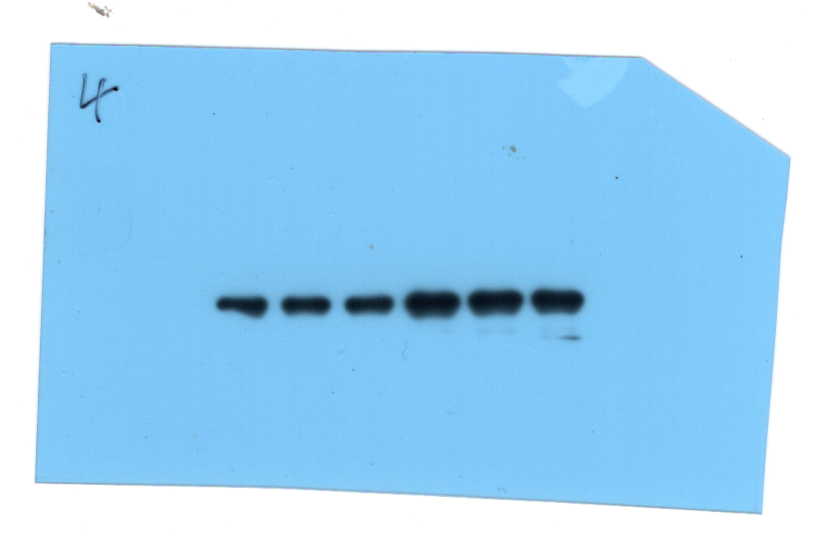

Supplement: Supplementary file 2 [file DataSheet1.ZIP › Original Source Data/Figure 8/Figure 8A-Western blot/MAP2K2-44kDa.tif]

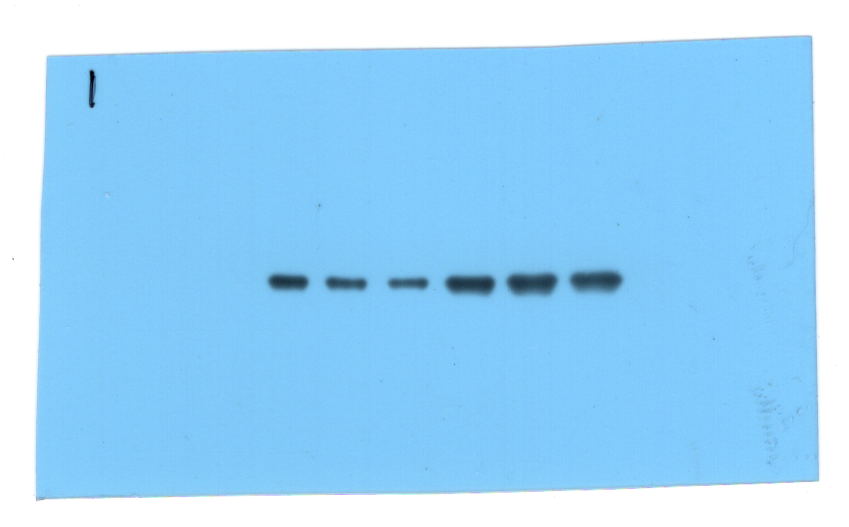

Supplement: Supplementary file 2 [file DataSheet1.ZIP › Original Source Data/Figure 8/Figure 8A-Western blot/MAP3K7-67kDa.tif]

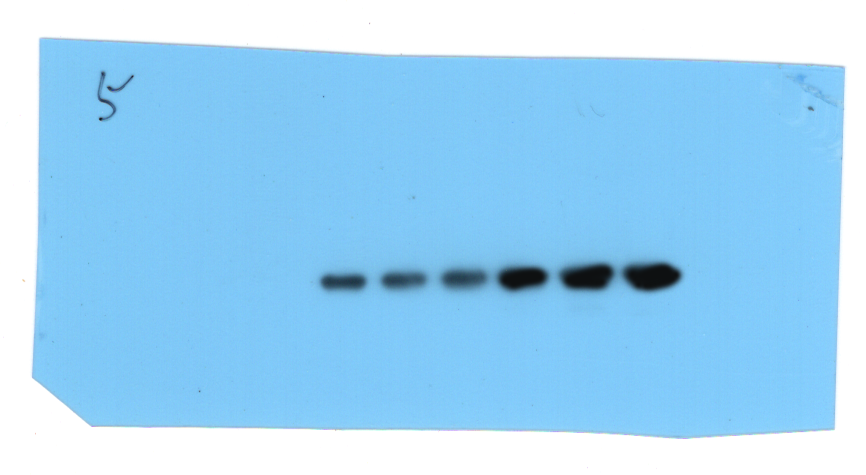

Supplement: Supplementary file 2 [file DataSheet1.ZIP › Original Source Data/Figure 8/Figure 8A-Western blot/RAC1-21kDa.tif]

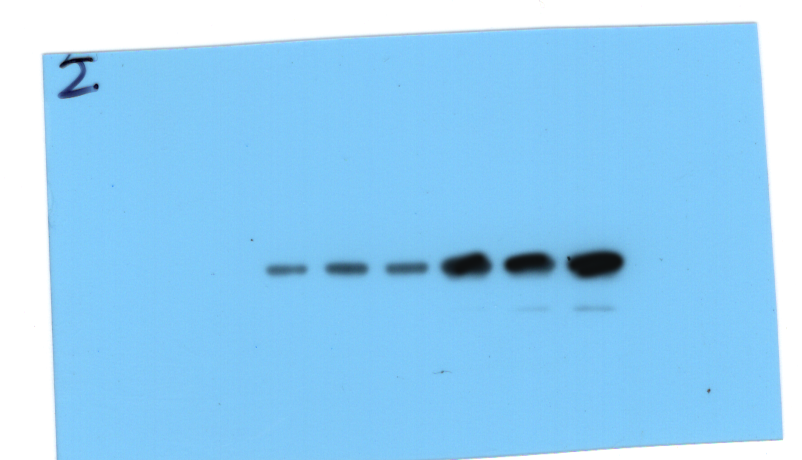

Supplement: Supplementary file 2 [file DataSheet1.ZIP › Original Source Data/Figure 8/Figure 8A-Western blot/SPP1-35kDa.tif]

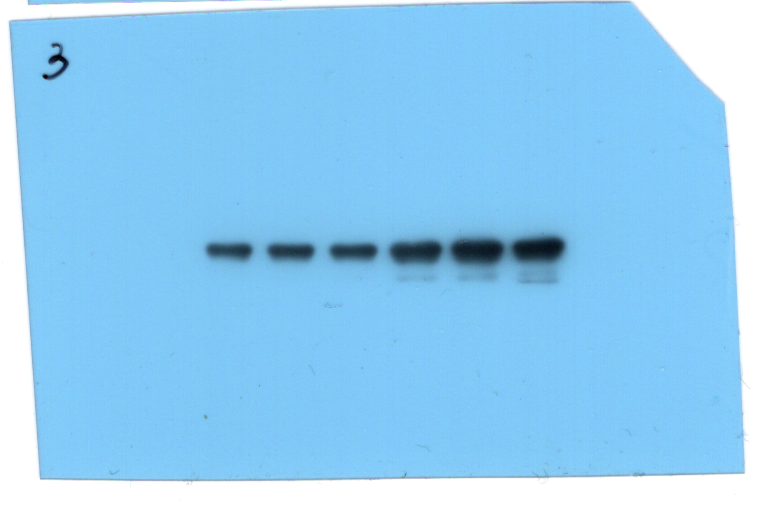

Supplement: Supplementary file 2 [file DataSheet1.ZIP › Original Source Data/Figure 8/Figure 8A-Western blot/TRAF3-64kDa.tif]

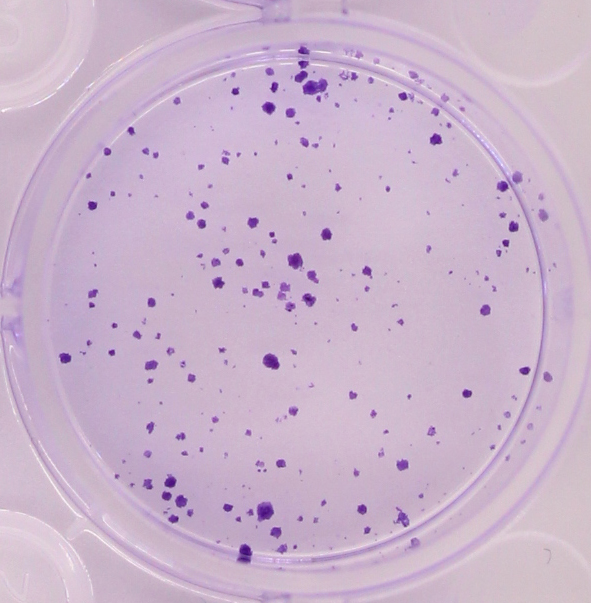

Supplement: Supplementary file 2 [file DataSheet1.ZIP › Original Source Data/Figure 9/Figure 9C-Clone formation assay/HepG2-si-MAP2K2#1.jpg]

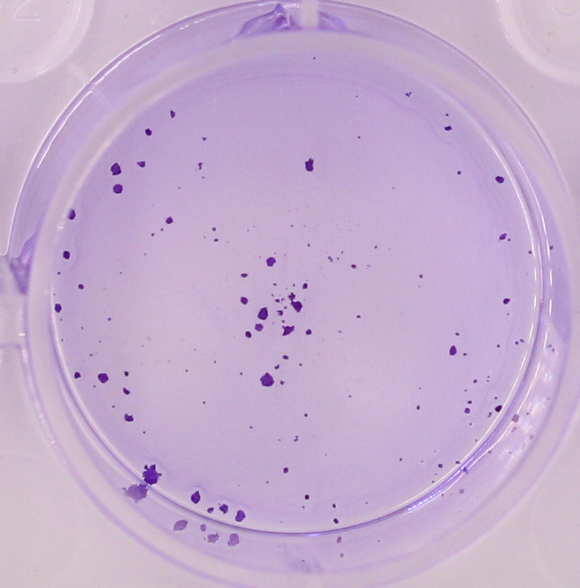

Supplement: Supplementary file 2 [file DataSheet1.ZIP › Original Source Data/Figure 9/Figure 9C-Clone formation assay/HepG2-si-MAP2K2#2.jpg]

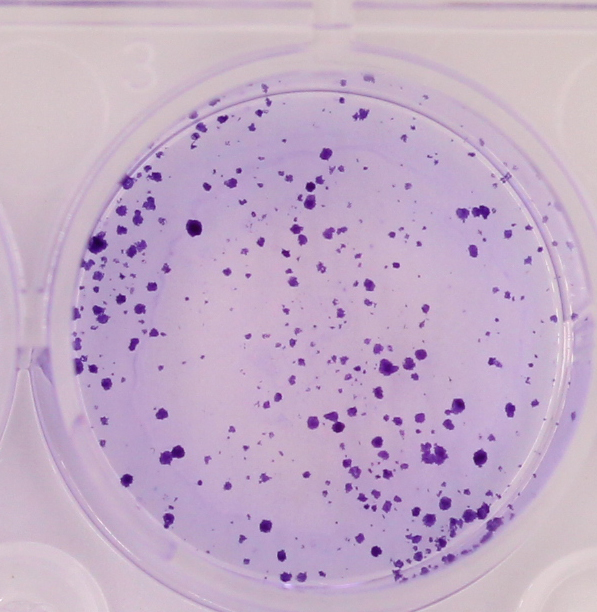

Supplement: Supplementary file 2 [file DataSheet1.ZIP › Original Source Data/Figure 9/Figure 9C-Clone formation assay/HepG2-si-NC.JPG]

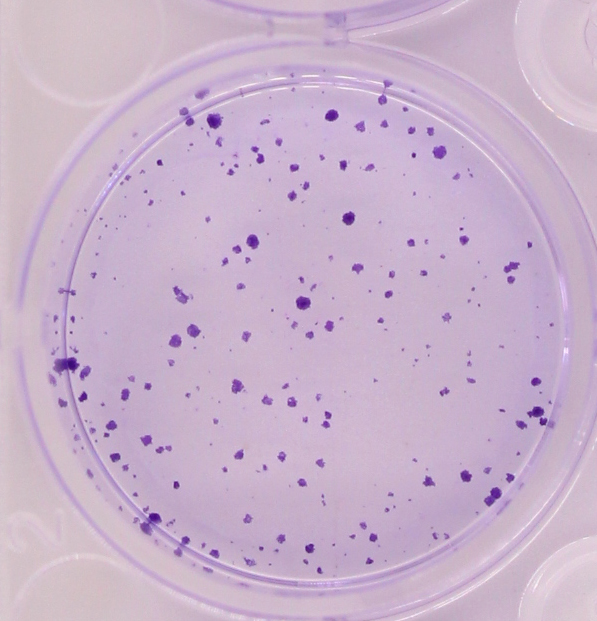

Supplement: Supplementary file 2 [file DataSheet1.ZIP › Original Source Data/Figure 9/Figure 9C-Clone formation assay/Huh7-si-MAP2K2#1.jpg]

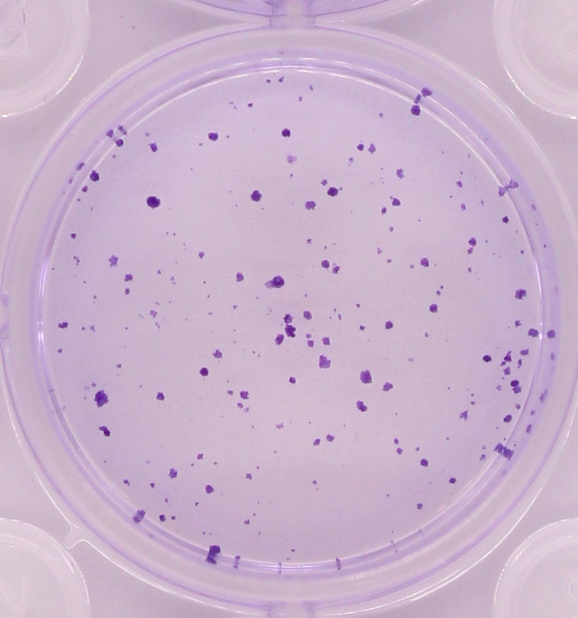

Supplement: Supplementary file 2 [file DataSheet1.ZIP › Original Source Data/Figure 9/Figure 9C-Clone formation assay/Huh7-si-MAP2K2#2.jpg]

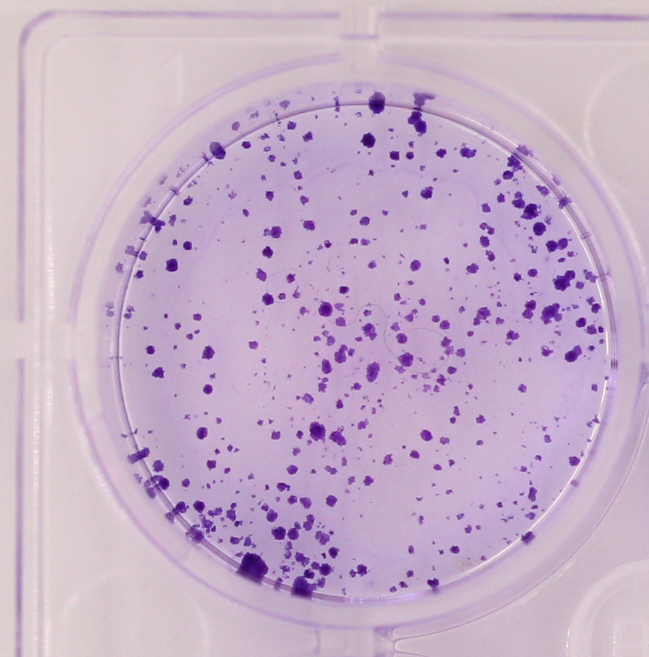

Supplement: Supplementary file 2 [file DataSheet1.ZIP › Original Source Data/Figure 9/Figure 9C-Clone formation assay/Huh7-si-NC.JPG]

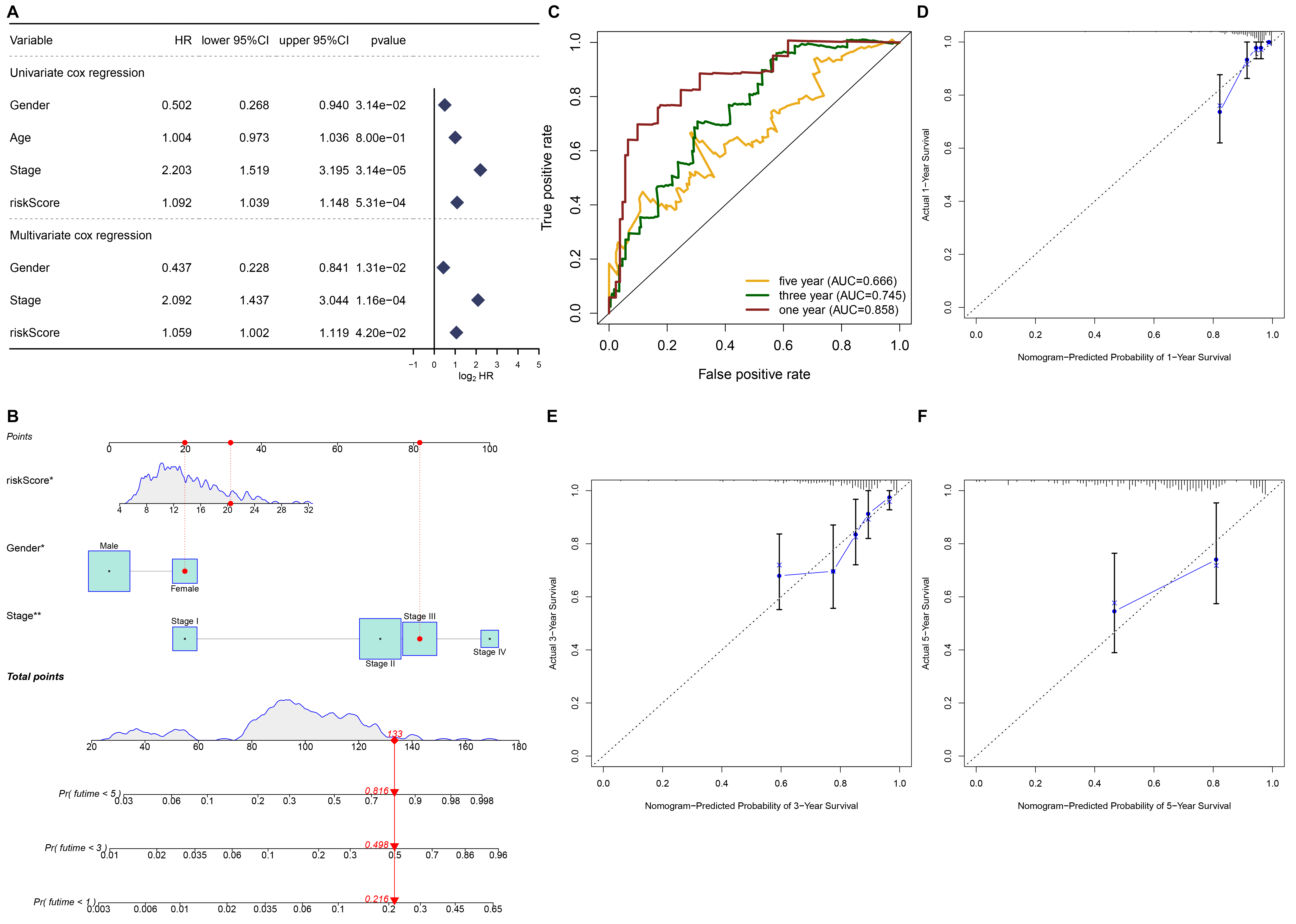

Supplement: Supplementary file 3 [file Image2.TIF]

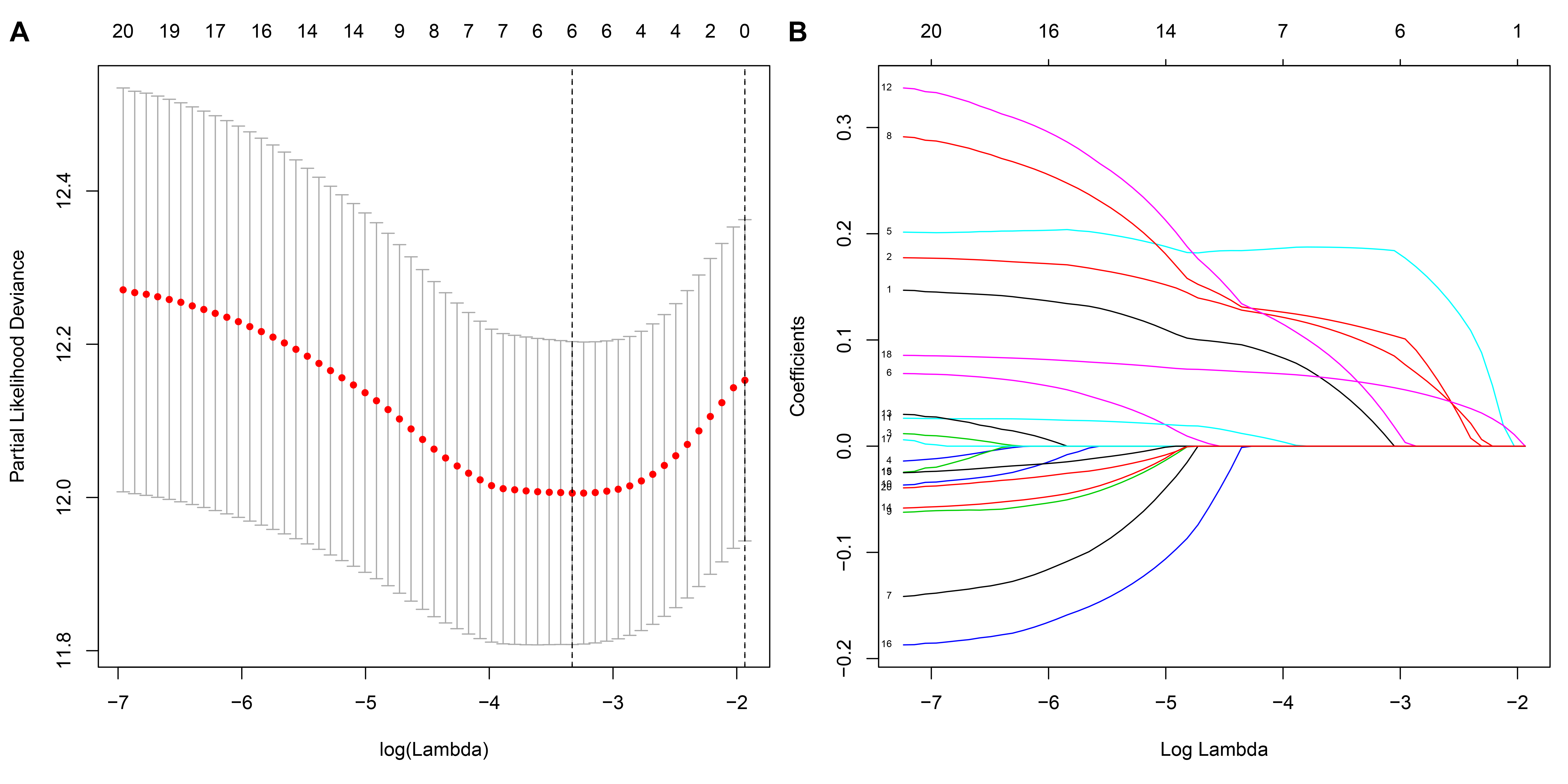

Supplement: Supplementary file 4 [file Image1.TIF]
