# Supplementary material for: Development of a Toll-Like Receptor-Based Gene Signature That Can Predict Prognosis, Tumor Microenvironment, and Chemotherapy Response for Hepatocellular Carcinoma
Source: Front Mol Biosci. 2021 Sep 21;8:729789. doi: 10.3389/fmolb.2021.729789 (PMC8490642; doi:10.3389/fmolb.2021.729789)
Supplement: Supplementary file 2 [file DataSheet1.ZIP › Original Source Data/Figure 9/Figure 9F-Flow cytometry/HepG2-si-MAP2K2#1.pdf]

标本19-33.63 报告

样本名：标本19-33.63　　　　仪器：BeamCyte
采样时间：N/A　　　　软件：CytoSYS 1.1

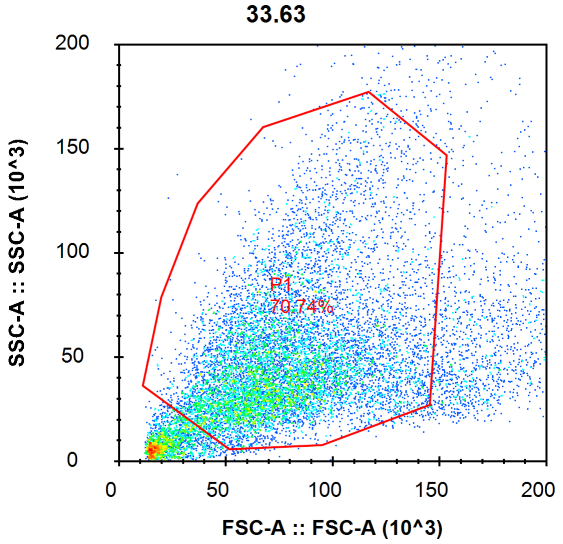

Gate	Count	%All	Mean X	Median X
All	14158	100.00%	90115	78866
P1	10016	70.74%	80373	77531

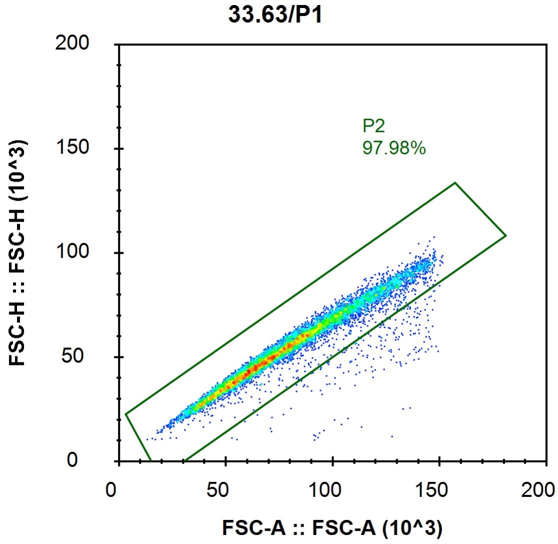

Gate	Count	%P1	Mean X	Median X
P1	10016	100.00%	80373	77531
P2	9814	97.98%	79551	76733

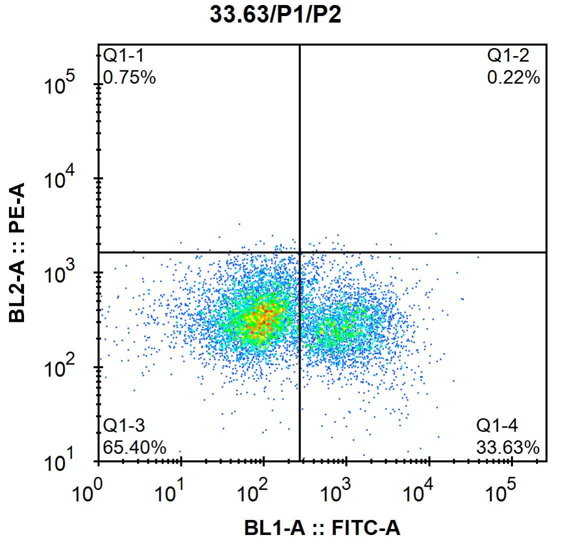

Gate	Count	%P2	Mean X	Median X
P2	9814	100.00%	702	122
Q1-1	74	0.75%	-81	-23
Q1-2	22	0.22%	1323	631
Q1-3	6418	65.40%	76	77
Q1-4	3300	33.63%	1932	953
