# Supplementary material for: Development of a Toll-Like Receptor-Based Gene Signature That Can Predict Prognosis, Tumor Microenvironment, and Chemotherapy Response for Hepatocellular Carcinoma
Source: Front Mol Biosci. 2021 Sep 21;8:729789. doi: 10.3389/fmolb.2021.729789 (PMC8490642; doi:10.3389/fmolb.2021.729789)
Supplement: Supplementary file 2 [file DataSheet1.ZIP › Original Source Data/Figure 9/Figure 9F-Flow cytometry/HepG2-si-MAP2K2#2.pdf]

标本19-44.34 报告

样本名：标本19-44.34
采样时间：N/A

仪器：BeamCyte
软件：CytoSYS 1.1

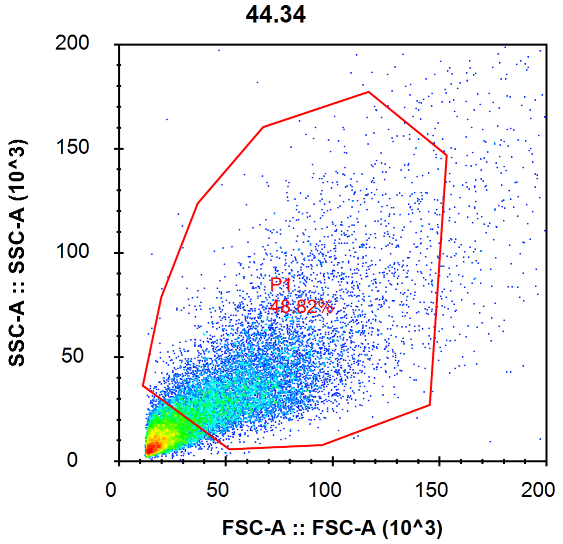

Gate	Count	%All	Mean X	Median X
All	20508	100.00%	48697	34194
P1	10013	48.82%	63568	59085

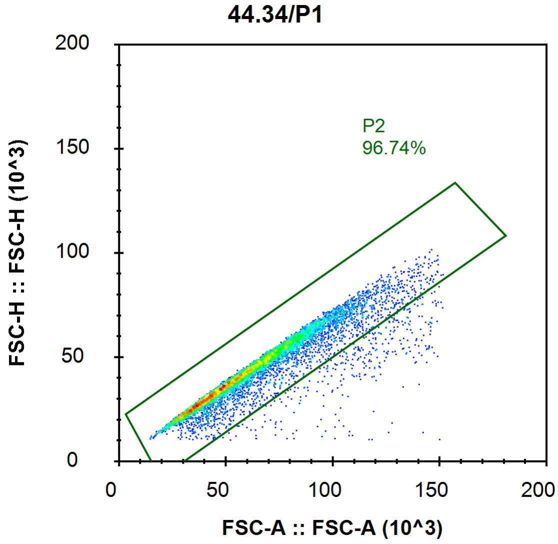

Gate	Count	%P1	Mean X	Median X
P1	10013	100.00%	63568	59085
P2	9687	96.74%	61907	58036

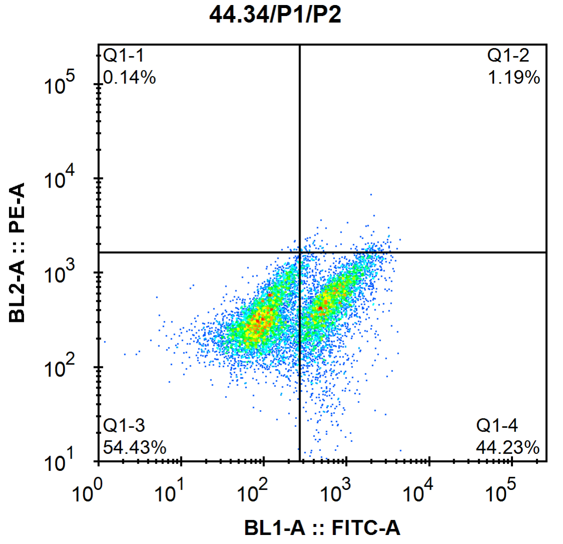

Gate	Count	%P2	Mean X	Median X
P2	9687	100.00%	439	211
Q1-1	14	0.14%	57	213
Q1-2	115	1.19%	1627	1824
Q1-3	5273	54.43%	110	99
Q1-4	4285	44.23%	813	625
