# Supplementary material for: Development of a Toll-Like Receptor-Based Gene Signature That Can Predict Prognosis, Tumor Microenvironment, and Chemotherapy Response for Hepatocellular Carcinoma
Source: Front Mol Biosci. 2021 Sep 21;8:729789. doi: 10.3389/fmolb.2021.729789 (PMC8490642; doi:10.3389/fmolb.2021.729789)
Supplement: Supplementary file 2 [file DataSheet1.ZIP › Original Source Data/Figure 9/Figure 9F-Flow cytometry/HepG2-si-NC.pdf]

标本19-9.33 报告

样本名：标本19-9.33
采样时间：N/A

仪器：BeamCyte
软件：CytoSYS 1.1

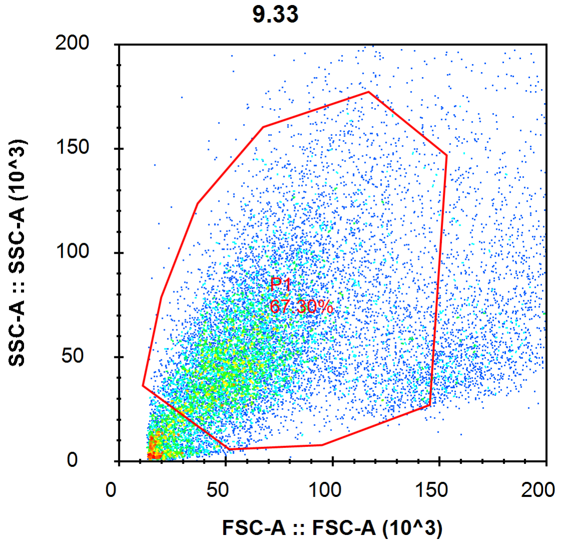

Gate	Count	%All	Mean X	Median X
All	16263	100.00%	88706	67462
P1	10945	67.30%	70606	64427

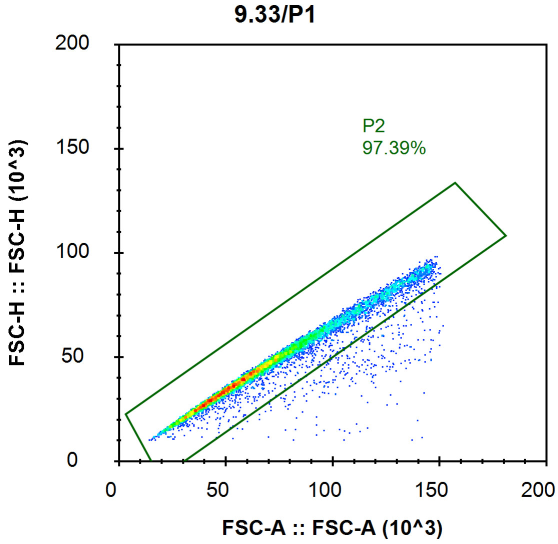

Gate	Count	%P1	Mean X	Median X
P1	10945	100.00%	70606	64427
P2	10659	97.39%	69425	63411

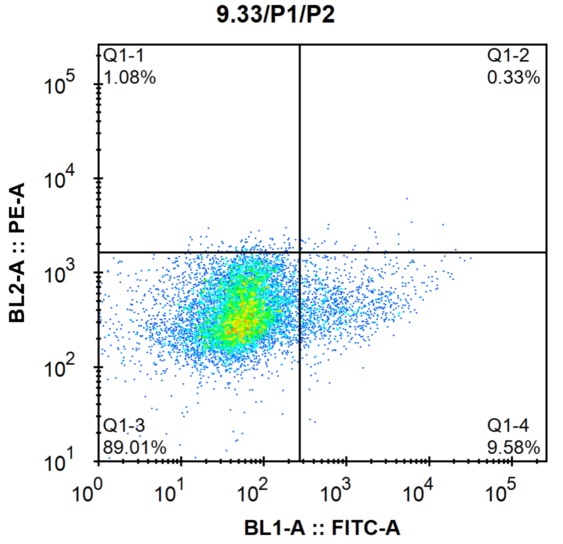

Gate	Count	%P2	Mean X	Median X
P2	10659	100.00%	211	53
Q1-1	115	1.08%	47	60
Q1-2	35	0.33%	2776	1205
Q1-3	9488	89.01%	56	48
Q1-4	1021	9.58%	1584	734
