# Supplementary material for: Development of a Toll-Like Receptor-Based Gene Signature That Can Predict Prognosis, Tumor Microenvironment, and Chemotherapy Response for Hepatocellular Carcinoma
Source: Front Mol Biosci. 2021 Sep 21;8:729789. doi: 10.3389/fmolb.2021.729789 (PMC8490642; doi:10.3389/fmolb.2021.729789)
Supplement: Supplementary file 2 [file DataSheet1.ZIP › Original Source Data/Figure 9/Figure 9F-Flow cytometry/Huh7-si-MAP2K2#1.pdf]

标本19-31.47 报告

样本名：标本19-31.47
采样时间：N/A

仪器：BeamCyte
软件：CytoSYS 1.1

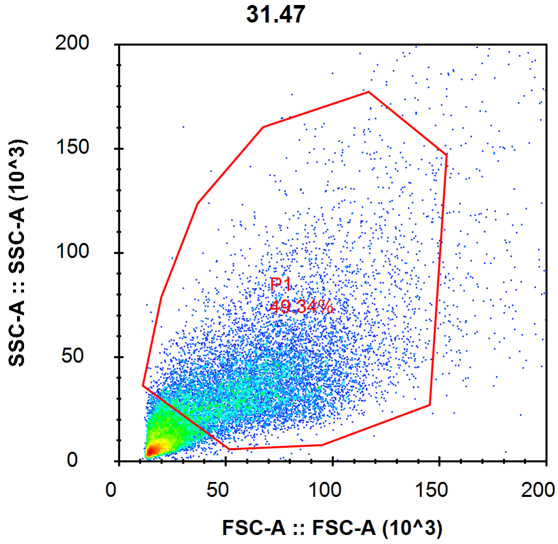

Gate	Count	%All	Mean X	Median X
All	20323	100.00%	48367	33168
P1	10028	49.34%	66236	63029

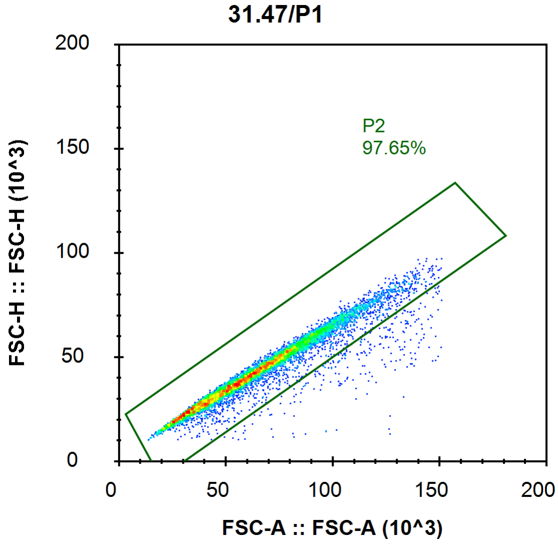

Gate	Count	%P1	Mean X	Median X
P1	10028	100.00%	66236	63029
P2	9792	97.65%	65081	62196

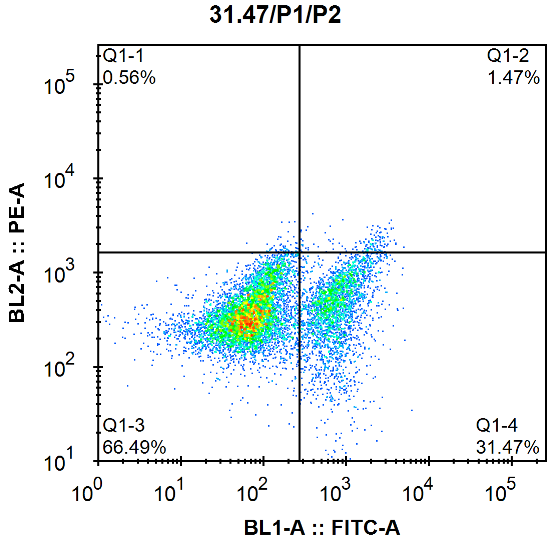

Gate	Count	%P2	Mean X	Median X
P2	9792	100.00%	397	106
Q1-1	55	0.56%	161	169
Q1-2	144	1.47%	1897	2040
Q1-3	6511	66.49%	78	69
Q1-4	3082	31.47%	1006	790
