# Supplementary material for: Development of a Toll-Like Receptor-Based Gene Signature That Can Predict Prognosis, Tumor Microenvironment, and Chemotherapy Response for Hepatocellular Carcinoma
Source: Front Mol Biosci. 2021 Sep 21;8:729789. doi: 10.3389/fmolb.2021.729789 (PMC8490642; doi:10.3389/fmolb.2021.729789)
Supplement: Supplementary file 2 [file DataSheet1.ZIP › Original Source Data/Figure 9/Figure 9F-Flow cytometry/Huh7-si-MAP2K2#2.pdf]

标本19-35.34 报告

样本名：标本19-35.34　　　　仪器：BeamCyte
采样时间：N/A　　　　软件：CytoSYS 1.1

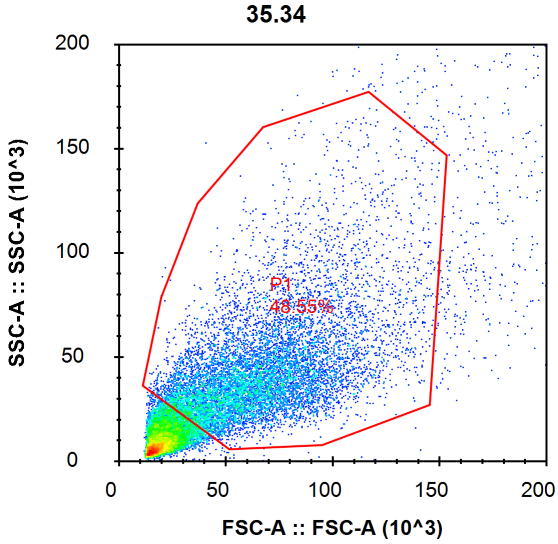

Gate	Count	%All	Mean X	Median X
All	20611	100.00%	50486	33689
P1	10007	48.55%	66623	63059

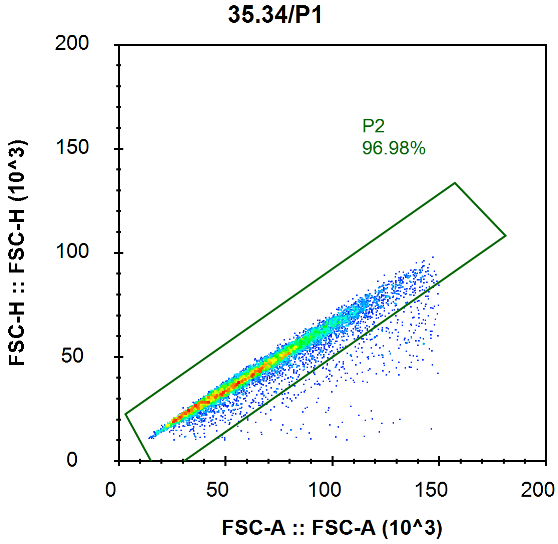

Gate	Count	%P1	Mean X	Median X
P1	10007	100.00%	66623	63059
P2	9705	96.98%	65156	62158

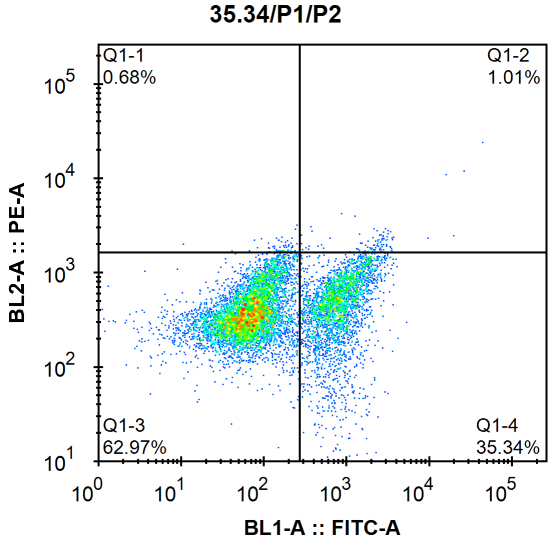

Gate	Count	%P2	Mean X	Median X
P2	9705	100.00%	461	113
Q1-1	66	0.68%	151	168
Q1-2	98	1.01%	3230	2389
Q1-3	6111	62.97%	77	68
Q1-4	3430	35.34%	1071	839
