# Supplementary material for: Development of a Toll-Like Receptor-Based Gene Signature That Can Predict Prognosis, Tumor Microenvironment, and Chemotherapy Response for Hepatocellular Carcinoma
Source: Front Mol Biosci. 2021 Sep 21;8:729789. doi: 10.3389/fmolb.2021.729789 (PMC8490642; doi:10.3389/fmolb.2021.729789)
Supplement: Supplementary file 2 [file DataSheet1.ZIP › Original Source Data/Figure 9/Figure 9F-Flow cytometry/Huh7-si-NC.pdf]

标本19-9.9 报告

样本名：标本19-9.9
采样时间：N/A

仪器：BeamCyte
软件：CytoSYS 1.1

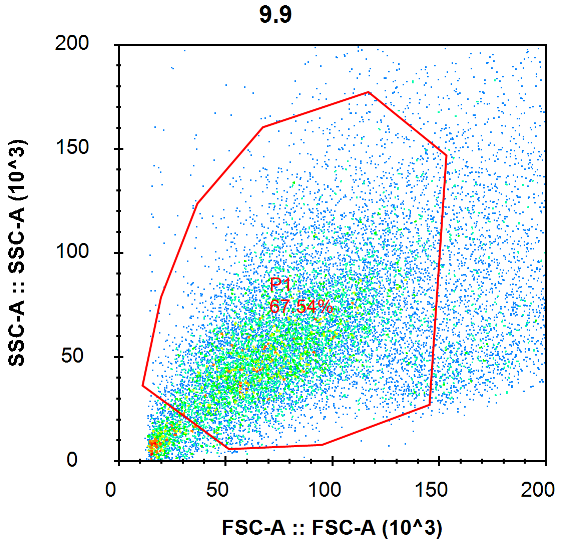

Gate	Count	%All	Mean X	Median X
All	16407	100.00%	107213	91731
P1	11082	67.54%	82959	80051

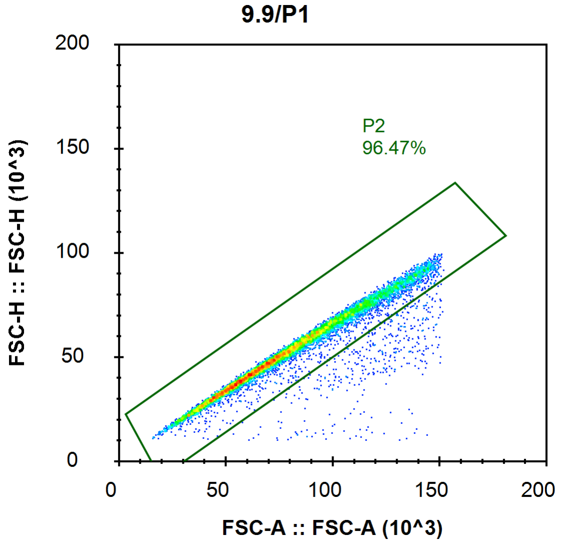

Gate	Count	%P1	Mean X	Median X
P1	11082	100.00%	82959	80051
P2	10691	96.47%	81605	78606

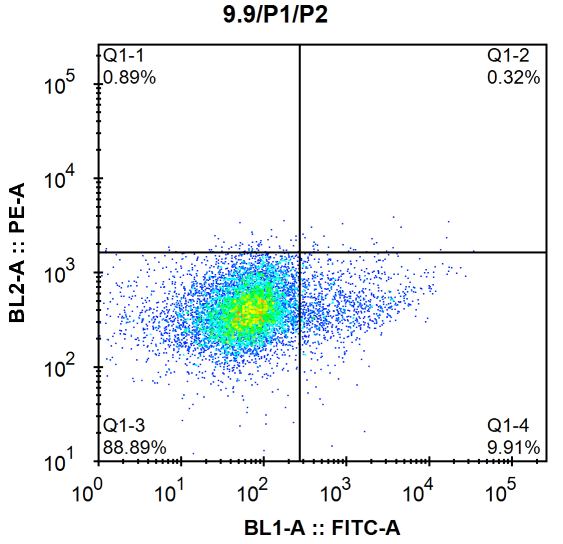

Gate	Count	%P2	Mean X	Median X
P2	10691	100.00%	227	62
Q1-1	95	0.89%	-262	38
Q1-2	34	0.32%	3618	1097
Q1-3	9503	88.89%	61	55
Q1-4	1059	9.91%	1650	782
