# Supplementary material for: Development of a Toll-Like Receptor-Based Gene Signature That Can Predict Prognosis, Tumor Microenvironment, and Chemotherapy Response for Hepatocellular Carcinoma
Source: Front Mol Biosci. 2021 Sep 21;8:729789. doi: 10.3389/fmolb.2021.729789 (PMC8490642; doi:10.3389/fmolb.2021.729789)
Supplement: Supplementary file 5 [file Table2.DOCX]

library(limma)

rt=read.table("symbol-TPM.txt",sep="\t",header=T,check.names=F)

rt=as.matrix(rt)

rownames(rt)=rt[,1]

exp=rt[,2:ncol(rt)]

dimnames=list(rownames(exp),colnames(exp))

data=matrix(as.numeric(as.matrix(exp)),nrow=nrow(exp),dimnames=dimnames)

data=avereps(data)

gene<-read.table("geneset.txt",head=F,sep='\t',check.names = F)

gene_expr<-data[intersect(gene$V1,row.names(data)),]

write.table(gene_expr,"gene_expr.txt",quote=F,sep='\t')

gene_expr=gene_expr[rowMeans(gene_expr)>0.5,]

logFoldChange=0.2

adjustP=0.05

conNum= 50

treatNum=374

rt=log2(gene_expr+1)

modType=c(rep("con",conNum),rep("treat",treatNum))

design <- model.matrix(~0+factor(modType))

colnames(design) <- c("con","treat")

fit <- lmFit(rt,design)

cont.matrix<-makeContrasts(treat-con,levels=design)

fit2 <- contrasts.fit(fit, cont.matrix)

fit2 <- eBayes(fit2)

allDiff=topTable(fit2,adjust='fdr',number=200000)

write.table(allDiff,file="mirnaAll.xls",sep="\t",quote=F)

#火山图

pdf(file="mirnaVol.pdf",height=5,width=5)

xMax=max(abs(allDiff$logFC))

yMax=max(-log10(allDiff$adj.P.Val))

plot(allDiff$logFC, -log10(allDiff$adj.P.Val), xlab="logFC",ylab="-log10(adj.P.Val)",

main="Volcano", ylim=c(0,36),xlim=c(-xMax,xMax),yaxs="i",pch=20, cex=0.8,col="grey60")

diffSub=subset(allDiff, adj.P.Val<adjustP & logFC>logFoldChange)

points(diffSub$logFC, -log10(diffSub$adj.P.Val), pch=20, col="#e84447",cex=0.8)

diffSub=subset(allDiff, adj.P.Val<adjustP & logFC<(-logFoldChange))

points(diffSub$logFC, -log10(diffSub$adj.P.Val), pch=20, col="#305a87",cex=0.8)

abline(v=0,lty=2,lwd=3)

dev.off()

library(pheatmap)

hmExp=rt[rownames(diffSig),]

Type=c(rep("Normal",conNum),rep("Tumor",treatNum))

names(Type)=colnames(rt)

Type=as.data.frame(Type)

diffSig <- allDiff[with(allDiff, (abs(logFC)>logFoldChange & adj.P.Val < adjustP )), ]

diffSigOut=rbind(id=colnames(diffSig),diffSig)

plotdata <- t(scale(t(hmExp)))

plotdata[plotdata > 3] <- 3

plotdata[plotdata < -3] <- -3

pheatmap(plotdata,

annotation=Type,

color = colorRampPalette(c("#3348a4", "#0b0b0c", "#ded633"))(199),

cluster_cols =F,

show_colnames = F,

scale="row",

fontsize = 10,

fontsize_row=10,

fontsize_col=10)

diff_expr<-rt[intersect(row.names(diffSig),row.names(rt)),]

write.table(t(diff_expr),"expTime.txt",quote=F,sep='\t')

library(survival)

pFilter=0.05

rt=read.table("expTime.txt",header=T,sep="\t",check.names=F,row.names=1)

outTab=data.frame()

sigGenes=c("futime","fustat")

for(i in colnames(rt[,3:ncol(rt)])){

cox <- coxph(Surv(futime, fustat) ~ rt[,i], data = rt)

coxSummary = summary(cox)

coxP=coxSummary$coefficients[,"Pr(>|z|)"]

if(coxP<pFilter){

sigGenes=c(sigGenes,i)

outTab=rbind(outTab,

cbind(id=i,

HR=coxSummary$conf.int[,"exp(coef)"],

HR.95L=coxSummary$conf.int[,"lower .95"],

HR.95H=coxSummary$conf.int[,"upper .95"],

pvalue=coxSummary$coefficients[,"Pr(>|z|)"])

)

}

}

write.table(outTab,file="uniCox.txt",sep="\t",row.names=F,quote=F)

uniSigExp=rt[,sigGenes]

uniSigExp=cbind(id=row.names(uniSigExp),uniSigExp)

write.table(uniSigExp,file="uniSigExp.txt",sep="\t",row.names=F,quote=F)

library("glmnet")

library("survival")

#LASSO

rt=read.table("uniSigExp.txt",header=T,sep="\t",row.names=1,check.names=F)

rt$futime=rt$futime/365

x=as.matrix(rt[,c(3:ncol(rt))])

y=data.matrix(Surv(rt$futime,rt$fustat))

fit <- glmnet(x, y, family = "cox")

pdf("lambda.pdf")

plot(fit, xvar = "lambda", label = TRUE)

dev.off()

cvfit <- cv.glmnet(x, y, family="cox", maxit = 1000)

pdf("cvfit.pdf")

plot(cvfit)

abline(v=log(c(cvfit$lambda.min,cvfit$lambda.1se)),lty="dashed")

dev.off()

coef <- coef(fit, s = cvfit$lambda.min)

index <- which(coef != 0)

actCoef <- coef[index]

lassoGene=row.names(coef)[index]

geneCoef=cbind(Gene=lassoGene,Coef=actCoef)

write.table(geneCoef,file="geneCoef.txt",sep="\t",quote=F,row.names=F)

riskScore=predict(cvfit, newx = x, s = "lambda.min",type="response")

outCol=c("futime","fustat",lassoGene)

risk=as.vector(ifelse(riskScore>median(riskScore),"high","low"))

outTab=cbind(rt[,outCol],riskScore=as.vector(riskScore),risk)

write.table(cbind(id=rownames(outTab),outTab),

file="trainRisk.txt",

sep="\t",

quote=F,

row.names=F)

#External validation

#ICGC

dat<-read.table("survivalInput.txt",head=T,sep='\t',check.names = F,row.names = 1)

expTime<-cbind(expTime[c(1,2)],log2(dat[,intersect(gene$V1,colnames(dat))]+1))

write.table(expTime,"expTime.txt",quote=F,sep='\t')

z<-expTime

s=as.matrix(z[,c(3:ncol(z))])

riskScore=predict(cvfit, newx = s, s = "lambda.min",type="response")

outCol=c("futime","fustat",lassoGene)

risk=as.vector(ifelse(riskScore>median(riskScore),"high","low"))

outTab=cbind(z[,outCol],riskScore=as.vector(riskScore),risk)

write.table(cbind(id=rownames(outTab),outTab),

file="testRisk.txt",

sep="\t",

quote=F,

row.names=F)

#GSE76427

z<-read.table("expTime.txt",head=T,sep='\t',check.names = F,row.names = 1)

s=as.matrix(z[,c(3:ncol(z))])

riskScore=predict(cvfit, newx = s, s = "lambda.min",type="response")

outCol=c("futime","fustat",lassoGene)

risk=as.vector(ifelse(riskScore>median(riskScore),"high","low"))

outTab=cbind(z[,outCol],riskScore=as.vector(riskScore),risk)

write.table(cbind(id=rownames(outTab),outTab),

file="testRisk.txt",

sep="\t",

quote=F,

row.names=F)

#GSE14520

z<-read.table("expTime.txt",head=T,sep='\t',check.names = F,row.names = 1)

s=as.matrix(z[,c(3:ncol(z))])

riskScore=predict(cvfit, newx = s, s = "lambda.min",type="response")

outCol=c("futime","fustat",lassoGene)

risk=as.vector(ifelse(riskScore>median(riskScore),"high","low"))

outTab=cbind(z[,outCol],riskScore=as.vector(riskScore),risk)

write.table(cbind(id=rownames(outTab),outTab),

file="testRisk.txt",

sep="\t",

quote=F,

row.names=F)

#Train dataset

#ROC

library(survivalROC)

rt=read.table("trainRisk.txt",header=T,sep="\t",check.names=F,row.names=1)

rocCol=c("#ebab1c","#006401","#8b2320")

aucText=c()

#5-year

pdf(file="Train_ROC.pdf",width=6,height=6)

par(oma=c(0.5,1,0,1),font.lab=1.5,font.axis=1.5)

roc=survivalROC(Stime=rt$futime, status=rt$fustat, marker = rt$riskScore, predict.time =5, method="KM")

plot(roc$FP, roc$TP, type="l", xlim=c(0,1), ylim=c(0,1),col=rocCol[1],

xlab="False positive rate", ylab="True positive rate",

lwd = 3, cex.main=1.3, cex.lab=1.2, cex.axis=1.2, font=1.2)

aucText=c(aucText,paste0("five year"," (AUC=",sprintf("%.3f",roc$AUC),")"))

abline(0,1)

#3-year

roc=survivalROC(Stime=rt$futime, status=rt$fustat, marker = rt$riskScore, predict.time =3, method="KM")

aucText=c(aucText,paste0("three year"," (AUC=",sprintf("%.3f",roc$AUC),")"))

lines(roc$FP, roc$TP, type="l", xlim=c(0,1), ylim=c(0,1),col=rocCol[2],lwd = 3)

#1-year

roc=survivalROC(Stime=rt$futime, status=rt$fustat, marker = rt$riskScore, predict.time =1, method="KM")

aucText=c(aucText,paste0("one year"," (AUC=",sprintf("%.3f",roc$AUC),")"))

lines(roc$FP, roc$TP, type="l", xlim=c(0,1), ylim=c(0,1),col=rocCol[3],lwd = 3)

legend("bottomright", aucText,lwd=3,bty="n",col=rocCol)

dev.off()

#survival curve

library(survival)

library("survminer")

rt=read.table("trainRisk.txt",header=T,sep="\t");

diff=survdiff(Surv(futime, fustat) ~risk,data = rt)

pValue=1-pchisq(diff$chisq,df=1)

pValue=signif(pValue,4)

pValue=format(pValue, scientific = TRUE)

fit <- survfit(Surv(futime, fustat) ~ risk, data = rt)

pdf(file="Train_survival.pdf",onefile = FALSE,

width = 5.5,

height =5)

ggsurvplot(fit,

data=rt,

conf.int=F,

pval=paste0("p=",pValue),

pval.size=4,

risk.table=TRUE,

legend.labs=c("High risk", "Low risk"),

legend.title="",

xlab="Time(years)",

break.time.by = 2,

risk.table.title="",

palette=c("#e95354", "#1b791c"),

risk.table.height=.30)

dev.off()

#GSE76427

library(survivalROC)

rt=read.table("testRisk.txt",header=T,sep="\t",check.names=F,row.names=1)

rocCol=c("#ebab1c","#006401","#8b2320")

aucText=c()

#5-year

pdf(file="Test_ROC.pdf",width=6,height=6)

par(oma=c(0.5,1,0,1),font.lab=1.5,font.axis=1.5)

roc=survivalROC(Stime=rt$futime, status=rt$fustat, marker = rt$riskScore, predict.time =5, method="KM")

plot(roc$FP, roc$TP, type="l", xlim=c(0,1), ylim=c(0,1),col=rocCol[1],

xlab="False positive rate", ylab="True positive rate",

lwd = 3, cex.main=1.3, cex.lab=1.2, cex.axis=1.2, font=1.2)

aucText=c(aucText,paste0("five year"," (AUC=",sprintf("%.3f",roc$AUC),")"))

abline(0,1)

#3-year

roc=survivalROC(Stime=rt$futime, status=rt$fustat, marker = rt$riskScore, predict.time =3, method="KM")

aucText=c(aucText,paste0("three year"," (AUC=",sprintf("%.3f",roc$AUC),")"))

lines(roc$FP, roc$TP, type="l", xlim=c(0,1), ylim=c(0,1),col=rocCol[2],lwd = 3)

#1-year

roc=survivalROC(Stime=rt$futime, status=rt$fustat, marker = rt$riskScore, predict.time =1, method="KM")

aucText=c(aucText,paste0("one year"," (AUC=",sprintf("%.3f",roc$AUC),")"))

lines(roc$FP, roc$TP, type="l", xlim=c(0,1), ylim=c(0,1),col=rocCol[3],lwd = 3)

legend("bottomright", aucText,lwd=3,bty="n",col=rocCol)

dev.off()

#survival curve

library(survival)

library("survminer")

rt=read.table("testRisk.txt",header=T,sep="\t");

diff=survdiff(Surv(futime, fustat) ~risk,data = rt)

pValue=1-pchisq(diff$chisq,df=1)

pValue=signif(pValue,4)

pValue=format(pValue, scientific = TRUE)

fit <- survfit(Surv(futime, fustat) ~ risk, data = rt)

Survival analysis

pdf(file="Test_survival.pdf",onefile = FALSE,

width = 5.5,

height =5)

ggsurvplot(fit,

data=rt,

conf.int=F,

pval=paste0("p=",pValue),

pval.size=4,

risk.table=TRUE,

legend.labs=c("High risk", "Low risk"),

legend.title="",

xlab="Time(years)",

break.time.by = 2,

risk.table.title="",

palette=c("#e95354", "#1b791c"),

risk.table.height=.30)

dev.off()

#GSE14520

library(survivalROC)

rt=read.table("testRisk.txt",header=T,sep="\t",check.names=F,row.names=1)

rocCol=c("#ebab1c","#006401","#8b2320")

aucText=c()

#5-year

pdf(file="Test_ROC.pdf",width=6,height=6)

par(oma=c(0.5,1,0,1),font.lab=1.5,font.axis=1.5)

roc=survivalROC(Stime=rt$futime, status=rt$fustat, marker = rt$riskScore, predict.time =5, method="KM")

plot(roc$FP, roc$TP, type="l", xlim=c(0,1), ylim=c(0,1),col=rocCol[1],

xlab="False positive rate", ylab="True positive rate",

lwd = 3, cex.main=1.3, cex.lab=1.2, cex.axis=1.2, font=1.2)

aucText=c(aucText,paste0("five year"," (AUC=",sprintf("%.3f",roc$AUC),")"))

abline(0,1)

#3-year

roc=survivalROC(Stime=rt$futime, status=rt$fustat, marker = rt$riskScore, predict.time =3, method="KM")

aucText=c(aucText,paste0("three year"," (AUC=",sprintf("%.3f",roc$AUC),")"))

lines(roc$FP, roc$TP, type="l", xlim=c(0,1), ylim=c(0,1),col=rocCol[2],lwd = 3)

#1-year

roc=survivalROC(Stime=rt$futime, status=rt$fustat, marker = rt$riskScore, predict.time =1, method="KM")

aucText=c(aucText,paste0("one year"," (AUC=",sprintf("%.3f",roc$AUC),")"))

lines(roc$FP, roc$TP, type="l", xlim=c(0,1), ylim=c(0,1),col=rocCol[3],lwd = 3)

legend("bottomright", aucText,lwd=3,bty="n",col=rocCol)

dev.off()

#survival curve

library(survival)

library("survminer")

rt=read.table("testRisk.txt",header=T,sep="\t");

diff=survdiff(Surv(futime, fustat) ~risk,data = rt)

pValue=1-pchisq(diff$chisq,df=1)

pValue=signif(pValue,4)

pValue=format(pValue, scientific = TRUE)

fit <- survfit(Surv(futime, fustat) ~ risk, data = rt)

Survival analysis

pdf(file="Test_survival.pdf",onefile = FALSE,

width = 5.5,

height =5)

ggsurvplot(fit,

data=rt,

conf.int=F,

pval=paste0("p=",pValue),

pval.size=4,

risk.table=TRUE,

legend.labs=c("High risk", "Low risk"),

legend.title="",

xlab="Time(years)",

break.time.by = 2,

risk.table.title="",

palette=c("#e95354", "#1b791c"),

risk.table.height=.30)

dev.off()

library(survival)

library(forestplot)

options(forestplot_new_page = FALSE)

clrs <- fpColors(box="green",line="darkblue", summary="royalblue")

rt=read.table("indepent.txt",header=T,sep="\t",check.names=F,row.names=1)

outTab=data.frame()

for(i in colnames(rt[,3:ncol(rt)])){

cox <- coxph(Surv(futime, fustat) ~ rt[,i], data = rt)

coxSummary = summary(cox)

coxP=coxSummary$coefficients[,"Pr(>|z|)"]

outTab=rbind(outTab,

cbind(id=i,

HR=coxSummary$conf.int[,"exp(coef)"],

HR.95L=coxSummary$conf.int[,"lower .95"],

HR.95H=coxSummary$conf.int[,"upper .95"],

pvalue=coxSummary$coefficients[,"Pr(>|z|)"])

)

}

write.table(outTab,file="uniCox.xls",sep="\t",row.names=F,quote=F)

library(survival)

library(forestplot)

options(forestplot_new_page = FALSE)

clrs <- fpColors(box="red",line="darkblue", summary="royalblue")

rt=read.table("Multi.txt",header=T,sep="\t",check.names=F,row.names=1)

multiCox=coxph(Surv(futime, fustat) ~ ., data = rt)

multiCoxSum=summary(multiCox)

outTab=data.frame()

outTab=cbind(

HR=multiCoxSum$conf.int[,"exp(coef)"],

HR.95L=multiCoxSum$conf.int[,"lower .95"],

HR.95H=multiCoxSum$conf.int[,"upper .95"],

pvalue=multiCoxSum$coefficients[,"Pr(>|z|)"])

outTab=cbind(id=row.names(outTab),outTab)

write.table(outTab,file="multiCox.xls",sep="\t",row.names=F,quote=F)

#Forestplot

mulcox<-read.table("multiCox.xls",head=T,sep='\t',check.names = F,stringsAsFactors = F)

unicox<-read.table("uniCox.xls",head=T,sep='\t',check.names = F,stringsAsFactors = F)

hrtable <- rbind(c("Univariate cox regression",NA,NA,NA,NA),

unicox,

c("Multivariate cox regression",NA,NA,NA,NA),

mulcox)

tabletext <- cbind(c("Variable",hrtable$variable),

c("HR",format(round(as.numeric(hrtable$HR),3),nsmall = 3)),

c("lower 95%CI",format(round(as.numeric(hrtable$lower.95CI),3),nsmall = 3)),

c("upper 95%CI",format(round(as.numeric(hrtable$upper.95CI),3),nsmall = 3)),

c("pvalue",formatC(as.numeric(hrtable$p), format = "e", digits = 2)))

tabletext

tabletext[2,] <- c("Univariate cox regression",NA,NA,NA,NA)

tabletext[8,] <- c("Multivariate cox regression",NA,NA,NA,NA)

forestplot(labeltext=tabletext,

mean=c(NA,as.numeric(hrtable$HR)),

lower=c(NA,as.numeric(hrtable$lower.95CI)),

upper=c(NA,as.numeric(hrtable$upper.95CI)),

graph.pos=6,

graphwidth = unit(.25,"npc"),

fn.ci_norm="fpDrawDiamondCI",

col=fpColors(box="#343b64", lines="#ffffff", zero = "black"),

boxsize=0.4,

lwd.ci=1,

ci.vertices.height = 0.1,ci.vertices=F,

zero=0,

lwd.zero=2,

xticks = c(-1,0,1,2,3,4,5),

lwd.xaxis=2,

xlab=expression("log"[2]~"HR"),

hrzl_lines=list("1" = gpar(lwd=2, col="black"),

"2" = gpar(lwd=1, col="grey50", lty=2),

"8" = gpar(lwd=1, col="grey50", lty=2),

"11" = gpar(lwd=2, col="black")),

txt_gp=fpTxtGp(label=gpar(cex=1.2),

ticks=gpar(cex=0.85),

xlab=gpar(cex=1),

title=gpar(cex=1.5)),

lineheight = unit(.75,"cm"),

colgap = unit(0.3,"cm"),

mar=unit(rep(1.5, times = 4), "cm"),

new_page = F

)

#Nomogram

library(survival)

library(regplot)

rt<-read.table("Nomogram.txt",head=T,sep='\t',check.names = F,row.names = 1)

res.cox=coxph(Surv(futime, fustat) ~ . , data = rt)

nom1<-regplot(res.cox,

plots = c("density", "boxes"),

clickable=F,

title="",

points=TRUE,

droplines=TRUE,

observation=rt[1,],

rank="sd",

failtime = c(1,3,5),

prfail = T)

pbc<-rt

pbc$died <- pbc$fustat==1

head(pbc)

library(rms)

dd<-datadist(pbc)

options(datadist="dd")

options(na.action="na.delete")

cox1 <- cph(Surv(futime,fustat) ~ gender + age + grade + stage + riskScore ,surv=T,x=T, y=T,time.inc = 1*365,data=pbc)

cal1<- calibrate(cox1, cmethod="KM", method="boot", u=1*365, m= 80, B=1000)

pdf("calibrate1.pdf")

plot(cal1,lwd=2,lty=1,errbar.col="black",xlim = c(0,1),ylim = c(0,1),xlab ="Nomogram-Predicted Probability of 1-Year Survival",ylab="Actual 1-Year Survival",col="blue",sub=F)

mtext("")

box(lwd = 0.5)

abline(0,1,lty = 3,lwd = 2,col = "black")

dev.off()

#3-year

cox3 <- cph(Surv(futime,fustat) ~ gender + age + grade + stage + riskScore ,surv=T,x=T, y=T,time.inc = 1*365*3,data=pbc)

cal3 <- calibrate(cox3, cmethod="KM", method="boot", u=1*365*3, m= 80, B=1000)

pdf("calibrate3.pdf")

plot(cal3,lwd=2,lty=1,errbar.col="black",xlim = c(0,1),ylim = c(0,1),xlab ="Nomogram-Predicted Probability of 3-Year Survival",ylab="Actual 3-Year Survival",col="blue",sub=F)

mtext("")

box(lwd = 0.5)

abline(0,1,lty = 3,lwd = 2,col = "black")

dev.off()

#5-year

cox5 <- cph(Surv(futime,fustat) ~ gender + age + grade + stage + riskScore ,surv=T,x=T, y=T,time.inc = 1*365*5,data=pbc)

cal5 <- calibrate(cox5, cmethod="KM", method="boot", u=1*365*5, m= 80, B=1000)

pdf("calibrate5.pdf")

plot(cal5,lwd=2,lty=1,errbar.col="black",xlim = c(0,1),ylim = c(0,1),xlab ="Nomogram-Predicted Probability of 5-Year Survival",ylab="Actual 5-Year Survival",col="blue",sub=F)

mtext("")

box(lwd = 0.5)

abline(0,1,lty = 3,lwd = 2,col = "black")

dev.off()

#ROC

rt<-read.table("Nomogram.txt",head=T,sep='\t',check.names = F,row.names = 1)

multiCox=coxph(Surv(futime, fustat) ~ ., data = rt)

multiCoxSum=summary(multiCox)

score=predict(multiCox, type="risk", newdata=rt)

outTab=cbind(rt, Nomogram=as.vector(score))

write.table(outTab,"Nomogram_risk.txt",quote=F,sep='\t')

library(survivalROC)

setwd("C:\\Users\\lexb4\\Desktop\\CGGA\\13.ROC")

rt=read.table("Nomogram_risk.txt",header=T,sep="\t",check.names=F,row.names=1)

rocCol=c("#ebab1c","#006401","#8b2320")

aucText=c()

#5-year

pdf(file="ROC.pdf",width=6,height=6)

par(oma=c(0.5,1,0,1),font.lab=1.5,font.axis=1.5)

roc=survivalROC(Stime=rt$futime, status=rt$fustat, marker = rt$Nomogram, predict.time =5, method="KM")

plot(roc$FP, roc$TP, type="l", xlim=c(0,1), ylim=c(0,1),col=rocCol[1],

xlab="False positive rate", ylab="True positive rate",

lwd = 3, cex.main=1.3, cex.lab=1.2, cex.axis=1.2, font=1.2)

aucText=c(aucText,paste0("five year"," (AUC=",sprintf("%.3f",roc$AUC),")"))

abline(0,1)

#3-year

roc=survivalROC(Stime=rt$futime, status=rt$fustat, marker = rt$Nomogram, predict.time =3, method="KM")

aucText=c(aucText,paste0("three year"," (AUC=",sprintf("%.3f",roc$AUC),")"))

lines(roc$FP, roc$TP, type="l", xlim=c(0,1), ylim=c(0,1),col=rocCol[2],lwd = 3)

#1-year

roc=survivalROC(Stime=rt$futime, status=rt$fustat, marker = rt$Nomogram, predict.time =1, method="KM")

aucText=c(aucText,paste0("one year"," (AUC=",sprintf("%.3f",roc$AUC),")"))

lines(roc$FP, roc$TP, type="l", xlim=c(0,1), ylim=c(0,1),col=rocCol[3],lwd = 3)

legend("bottomright", aucText,lwd=3,bty="n",col=rocCol)

dev.off()

library(survival)

library(survminer)

risk=read.table("risk.txt",header=T,sep="\t",check.names=F,row.names=1)

cli=read.table("clinical.txt",sep="\t",check.names=F,header=T,row.names=1)

sameSample=intersect(row.names(cli),row.names(risk))

risk=risk[sameSample,]

cli=cli[sameSample,]

data=cbind(futime=risk[,1],fustat=risk[,2],cli,risk=risk[,"risk"])

for(i in colnames(data[,3:(ncol(data)-1)])){

rt=data[,c("futime","fustat",i,"risk")]

rt=rt[(rt[,i]!="unknow"),]

colnames(rt)=c("futime","fustat","clinical","risk")

tab=table(rt[,"clinical"])

tab=tab[tab!=0]

for(j in names(tab)){

rt1=rt[(rt[,"clinical"]==j),]

tab1=table(rt1[,"risk"])

tab1=tab1[tab1!=0]

labels=paste0(names(tab1)," risk(n=",tab1,")")

if(length(labels)==2){

titleName=j

if((i=="age") | (i=="Age") | (i=="AGE")){

titleName=paste0("age",j)

}

diff=survdiff(Surv(futime, fustat) ~risk,data = rt1)

pValue=1-pchisq(diff$chisq,df=1)

if(pValue<0.001){

pValue="p<0.001"

}else{

pValue=paste0("p=",sprintf("%.03f",pValue))

}

fit <- survfit(Surv(futime, fustat) ~ risk, data = rt1)

surPlot=ggsurvplot(fit,

data=rt1,

pval=pValue,

pval.size=6,

legend.labs=labels,

legend.title=titleName,

font.legend=13,

xlab="Time(years)",

break.time.by = 1,

palette=c("#e95354", "#1b791c") )

j=gsub(">=","ge",j);j=gsub("<=","le",j);j=gsub(">","gt",j);j=gsub("<","lt",j)

pdf(file=paste0("survival.",i,"_",j,".pdf"),onefile = FALSE,

width = 6,

height =6)

print(surPlot)

dev.off()

}

}

}

#GSEA

library(plyr)

library(ggplot2)

library(grid)

library(gridExtra)

setwd("D:\\biowolf\\metabolism\\24.multipleGSEA")

files=grep(".xls",dir(),value=T)

data = lapply(files,read.delim)

names(data) = files

dataSet = ldply(data, data.frame)

dataSet$pathway = gsub(".xls","",dataSet$.id)

gseaCol=c("#58CDD9","#6E568C","#E0367A","#D8D155","#64495D","#7CC767","#223D6C","#D20A13","#FFD121","#088247","#11AA4D")

pGsea=ggplot(dataSet,aes(x=RANK.IN.GENE.LIST,y=RUNNING.ES,fill=pathway,group=pathway))+

geom_point(shape=21) + scale_fill_manual(values = gseaCol[1:nrow(dataSet)]) +

labs(x = "", y = "Enrichment Score", title = "") + scale_x_continuous(expand = c(0, 0)) +

scale_y_continuous(expand = c(0, 0),limits =c(min(dataSet$RUNNING.ES-0.02), max(dataSet$RUNNING.ES+0.02))) +

theme_bw() + theme(panel.grid =element_blank()) + theme(panel.border = element_blank()) +

theme(axis.line = element_line(colour = "black")) + theme(axis.line.x = element_blank(),axis.ticks.x = element_blank(),axis.text.x = element_blank()) +

geom_hline(yintercept = 0) + guides(fill=guide_legend(title = NULL)) +

theme(legend.background = element_blank()) + theme(legend.key = element_blank())

pGene=ggplot(dataSet,aes(RANK.IN.GENE.LIST,pathway,colour=pathway))+geom_tile()+

scale_color_manual(values = gseaCol[1:nrow(dataSet)]) +

labs(x = "High risk<----------->Low risk", y = "", title = "") +

scale_x_discrete(expand = c(0, 0)) + scale_y_discrete(expand = c(0, 0)) +

theme_bw() + theme(panel.grid = element_blank()) + theme(panel.border = element_blank()) + theme(axis.line = element_line(colour = "black"))+

theme(axis.line.y = element_blank(),axis.ticks.y = element_blank(),axis.text.y = element_blank())+ guides(color=FALSE)

gGsea = ggplot_gtable(ggplot_build(pGsea))

gGene = ggplot_gtable(ggplot_build(pGene))

maxWidth = grid::unit.pmax(gGsea$widths, gGene$widths)

gGsea$widths = as.list(maxWidth)

gGene$widths = as.list(maxWidth)

dev.off()

pdf('multipleGSEA.pdf',

width=9,

height=5)

par(mar=c(5,5,2,5))

grid.arrange(arrangeGrob(gGsea,gGene,nrow=2,heights=c(.8,.3)))

dev.off()

#ssGSEA enrichment

gmtFile="immune.gmt"

library(GSVA)

library(limma)

library(GSEABase)

mat<-read.table("uniq_symbol.txt",head=T,sep='\t',check.names = F,row.names = 1)

mat<-as.matrix(mat)

geneSet=getGmt(gmtFile,

geneIdType=SymbolIdentifier())

ssgseaScore=gsva(mat, geneSet, method='ssgsea', kcdf='Gaussian', abs.ranking=TRUE)

write.table(t(ssgseaScore),"ssGSEAscore.txt",quote=F,sep='\t')

df<-read.table("GSEA_score.txt",head=T,sep='\t',check.names = F,row.names = 1)

data<-melt(df,

id.vars = c('Risk'),

measure.vars = colnames(df[-1]),

variable.name='Immune_check',

value.name='Expression')

p=ggboxplot(data, x="Immune_check", y="Expression", fill = "Risk",

ylab="Enirch score",

xlab="",

palette = c("aaas") )

p=p+rotate_x_text(60)

p+stat_compare_means(aes(group=Risk),symnum.args=list(cutpoints = c(0, 0.001, 0.01, 0.05, 1), symbols = c("***", "**", "*", "ns")),label = "p.signif")+theme(axis.text = element_text(size = 13, face = "bold"))

#Estimate

library(estimate)

filterCommonGenes(input.f="uniq_symbol.txt",

output.f="commonGenes.gct",

id="GeneSymbol")

estimateScore(input.ds = "commonGenes.gct",

output.ds="estimateScore.gct")

scores=read.table("estimateScore.gct",skip = 2,header = T)

rownames(scores)=scores[,1]

scores=t(scores[,3:ncol(scores)])

rownames(scores)=gsub("\\.","\\-",rownames(scores))

out=rbind(ID=colnames(scores),scores)

write.table(out,file="Estimate_scores.txt",sep="\t",quote=F,col.names=F)

#Correlation with immune cell

library(limma)

library(ggplot2)

library(ggpubr)

library(ggExtra)

immune=read.table("GSEA_score.txt",sep="\t",header=T,row.names=1,check.names=F)

immune[1:4,1:4]

immune1<-immune

rt<-read.table("Trainrisk.txt",head=T,sep='\t',che)

rt<-read.table("Trainrisk.txt",head=T,sep='\t',check.names = F,row.names = 1)

head(rt)

x<-as.numeric(rt$riskScore)

head(x)

outTab=data.frame()

for(j in colnames(immune1)[1:28]){

y=as.numeric(immune1[,j])

if(sd(y)>0){

df1=as.data.frame(cbind(x,y))

corT=cor.test(x,y,method="spearman")

cor=corT$estimate

pValue=corT$p.value

p1=ggplot(df1, aes(x, y)) +

ylab(j)+xlab("riskScore")+

geom_point()+ geom_smooth(method="lm",formula=y~x) + theme_bw()+

stat_cor(method = 'spearman', aes(x =x, y =y))

if(pValue<0.05){

pdf(file=paste0(j,".pdf"),width=5,height=5)

print(p1)

dev.off()

outTab=rbind(outTab,cbind(Cell=j,pValue))

}

}

}

write.table(outTab,file="immuneCor.result.txt",sep="\t",row.names=F,quote=F)

#Drug sensitivity Prediction

dat <- read.table("uniq_symbol.txt",sep = "\t",row.names = 1,header = T,stringsAsFactors = F,check.names = F)

ann <- read.table("Trainrisk.txt",sep = "\t",row.names = 1,header = T,stringsAsFactors = F,check.names = F)

library(pRRophetic)

## Warning: replacing previous import 'car::Anova' by 'genefilter::Anova' when

## loading 'pRRophetic'

library(ggplot2)

library(cowplot)

Sys.setenv(LANGUAGE = "en")

options(stringsAsFactors = FALSE)

GCP.drug <- read.table("Drug.txt")

GCP.drug <- GCP.drug$V1

jco <- c("#EABF00", "#2874C5", "red")

GCPinfo <- GCP.IC50 <- GCP.expr <- cvOut <- predictedPtype <- predictedBoxdat <- list()

plotp <- list()

for (drug in GCP.drug) {

set.seed(1248103)

cat(drug," starts!\n")

predictedPtype[[drug]] <- pRRopheticPredict(testMatrix = as.matrix(dat[,rownames(ann)]),

drug = drug,

tissueType = "allSolidTumors",

selection = 1)

if(!all(names(predictedPtype[[drug]])==rownames(ann))) {stop("Name mismatched!\n")}

predictedBoxdat[[drug]] <- data.frame("est.ic50"=predictedPtype[[drug]],

"risk"=ann$risk,

row.names = names(predictedPtype[[drug]]))

predictedBoxdat[[drug]]$risk <- factor(predictedBoxdat[[drug]]$risk,levels = c("high","low"),ordered = T)

p <- ggplot(data = predictedBoxdat[[drug]], aes(x=risk, y=est.ic50))

p <- p + geom_boxplot(aes(fill = risk)) +

scale_fill_manual(values = jco[1:length(unique(ann$risk))]) +

theme(legend.position="none") +

theme(axis.text.x = element_text(angle = 45, hjust = 1,size = 12),plot.title = element_text(size = 12, hjust = 0.5)) +

xlab("") + ylab("Estimated IC50") +

ggtitle(drug)

plotp[[drug]] <- p

cat(drug," has been finished!\n")

}

ggsave("boxplot of predicted IC50.pdf", width = 6, height = 5)

p <- vector()

for (drug in GCP.drug) {

tmp <- wilcox.test(as.numeric(predictedBoxdat[[drug]][which(predictedBoxdat[[drug]]$risk %in% "high"),"est.ic50"]),

as.numeric(predictedBoxdat[[drug]][which(predictedBoxdat[[drug]]$risk %in% "low"),"est.ic50"]),alternative = "less")$p.value

p <- append(p,tmp)

}

names(p) <- GCP.drug

print(p)

write.table(p,"output_pvalue.txt", quote = F, sep = "\t")
